# Biochemical Composition and Energy Strategy Along the Reproductive Cycle of Female *Octopus vulgaris* in Galician Waters (NW Spain)

**DOI:** 10.3389/fphys.2020.00760

**Published:** 2020-07-15

**Authors:** Pilar Sieiro, Jaime Otero, Santiago P. Aubourg

**Affiliations:** ^1^Campus do Mar (Doctoral Program Do*MAR), Universidade de Vigo, Vigo, Spain; ^2^Departamento de Oceanografía, Instituto de Investigaciones Marinas (CSIC), Vigo, Spain; ^3^Departamento de Tecnología de Alimentos, Instituto de Investigaciones Marinas (CSIC), Vigo, Spain

**Keywords:** reproduction, lipids, fatty acids, proteins, glycogen, energy allocation, *Octopus vulgaris*, NE Atlantic Ocean

## Abstract

The common octopus, *Octopus vulgaris*, has a short life cycle, growing rapidly to maturity, spawning once, and characterized by an asynchronic oocyte development and a synchronic ovulation dying after breeding. This species has a protein and amino acid metabolism and it is usually defined as an income breeder. However, most biochemical studies lack an examination of the whole reproductive cycle, in particular the spawning process. We here studied the biochemical changes and determined the energy strategy along reproduction in female *O. vulgaris*, and found that proteins were the main energy reserve, primarily located in the body muscle when sexually maturing and decreasing during breeding. Lipids were also an important source of energy in the ovary and digestive gland and decreased during breeding too. By contrast, glycogen had a minor contribution to the energy content and was the unique compound that increased in spawning and post-spawning females. Additionally, the most abundant fatty acids (FA) in all tissues were 16:0, 18:0, 20:1n9, 20:4n6 (ARA), 20:5n3 (EPA) and 22:6n3 (DHA), with a clear predominance of long-chain polyunsaturated FA. The FA profile of mature ovaries was compared with other life stages finding similitudes with eggs, hatchlings and juveniles but considerable differences with paralarvae which showed higher DHA/ARA and EPA/ARA ratios. Therefore, we found important biochemical changes along the reproductive cycle that determined the energetic signature in each tissue, though no significant energy trade-offs between tissues were found, suggesting that, on the one hand, female *O. vulgaris* obtained energy directly from food accumulated simultaneously in the somatic and reproductive tissues during sexual maturation. However, an energy reallocation from somatic to reproductive growth would occur once vitellogenesis has started, so that the rate at which body growths would decrease in favor of ovary growth. On the other hand, during breeding, a general decrease in the energy content occurred in all tissues, so that the ovary would be responsible for the spawning success, whereas muscle tissues and digestive gland would independently supply the energy needed for the body maintenance safeguarding the female survival needed for the maternal care.

## Introduction

For many marine invertebrates, like in other animals, reproduction is the most energy intensive period of their life, having to optimize the limited energy inputs from food for both maintenance of somatic structures and the reproductive investments (Calow, 1981; Roff, 2002). In general, there is a negative correlation between the amount of energy invested in reproduction at one time and the subsequent survival and/or reproductive performance of the parent (Calow, 1979), so that species investing most of their energy for reproduction will compromise their body condition, spawning only once in their lives, and reducing their life-span (called semelparous with terminal spawning; Rocha et al., 2001). On the contrary, those species who save some energy for survival, and possibly further growing, will spawn more than one time (called iteroparous with or without terminal spawning). Once maturation starts, there is often a decline in energy reserves or energy availability, which can be described as a cost. This cost can be either sourced from energy acquired and stored previously during a period of food abundance and drawing on it later for reproduction (capital breeders), or directly acquired from locally ingested food and allocating it rapidly to reproduction (income breeders) (Jönsson, 1997). Typically, this reproductive investment in gonad tissues and the fluctuation in somatic condition are largely studied in females as compared to males and usually in conjunction with effects of abiotic factors on growth, such as temperature or food availability, since they might have an impact on the timing and level of energy diverted to reproduction (e.g., Domínguez-Petit and Saborido-Rey, 2010; Lourenço et al., 2014; Lin et al., 2017).

Both reproductive strategies, capital and income breeding, have been generally described in multiple fish species: capital breeders rely on an accumulation of energy prior to egg production with a storage of lipids in the somatic tissues or the liver that will be later used for reproduction (Zaboukas et al., 2006; Alonso-Fernández and Saborido-Rey, 2012; Villegas-Ríos et al., 2014); whereas income breeders depend more on the environmental conditions and food availability during the spawning season than on body energy reserves (Luo and Musick, 1991; Domínguez-Petit and Saborido-Rey, 2010). Both type of breeders represent the extremes of a continuum and certain species may compensate for inadequate energy deposits with a direct income derived from feeding (Henderson et al., 1996). Therefore, an individual’s position along this strategic breeding continuum may shift along ontogeny or with environmental conditions (McBride et al., 2015). In marine invertebrates, strategies of energy accumulation may vary among groups of taxa. For instance, bivalve mollusks store glycogen in somatic tissues and in some cases in the digestive gland (e.g., Galap et al., 1997; Barber and Blake, 2006), whereas in crustaceans and gastropod mollusks, the general idea is that proteins, lipids or glycogen in the digestive gland and/or proteins or glycogen in muscle tissues are devoted to gonad maturation (Lambert and Dehnel, 1974; Najmudeen, 2007; Yan et al., 2017). The metabolism of cephalopods is mainly based on proteins and, therefore, a high amino acid requirement exists in order to maintain optimal growth and energy supply (Lee, 1994). In fact, proteins are the major constituent of the tissues of several male cephalopods, representing between 30 and >60% in dry weight, whereas lipids and glycogen are found in amounts of <5 and 55%, and <2% and 12, respectively, depending on the tissue and the species (Rosa et al., 2005b). Furthermore, cephalopods tend to have a limited ability to digest lipids, and therefore they are stored and used during fasting or even mobilized for energy or gonad development. By contrast, glycogen reserves use to be utilized during resting metabolism and burst activity (O’Dor and Webber, 1986).

The energetic cost of reproduction can be measured at various levels: whole animal, cellular organization, or tissue composition, with most authors having studied this cost of reproduction in cephalopods through morphometric analyses (e.g., Collins et al., 1995; Ho et al., 2004; Otero et al., 2007; Smith et al., 2011; Lourenço et al., 2012; Lin et al., 2015), and just a few focusing on studying changes in the structural composition (e.g., Moltschaniwskyj, 1995; Moltschaniwskyj and Carter, 2013) or through detailed biochemical analyses (e.g., de Moreno et al., 1998; Moltschaniwskyj and Semmens, 2000). Structural and/or biochemical analyses throughout maturation unlike morphometric analysis are more desirable because they offer a more detailed knowledge of patterns of energy storage and utilization to gain a comprehensive understanding of the interaction between the organism and its environment, and may reveal where constraints of power budgeting are most critical (Clarke et al., 1994). However, most analyses on biochemical changes throughout reproduction in cephalopods suffer from the lack of information during post-spawning, and this is more pervasive for those species, such as some octopods, which hide for parenting. Nonetheless, some studies have dealt with the biochemistry of maturation in a number of female cephalopod species finding some common patterns such as the accumulation of lipids and/or proteins in the ovary (e.g., Jackson et al., 2004), and a stable composition in the mantle, particularly with regard to proteins, during maturation (Clarke et al., 1994; de Moreno et al., 1998; Moltschaniwskyj and Semmens, 2000; Moltschaniwskyj and Carter, 2013). Regarding octopods, those particularities outlined previously are also common, for instance, the accumulation of lipids in the ovary (Pollero and Iribarne, 1988; Rosa et al., 2004b) and in the digestive gland in some cases (Lourenço et al., 2014; Pascual et al., 2020). However, there are several other points of dissent such as the absence or not of any variation in the protein content in the digestive gland and the ovary (Rosa et al., 2004a, b), or the differences regarding the content of glycogen in any of these tissues (Olivares et al., 2019; Pascual et al., 2020). Finally, during spawning and post-spawning stages it is frequently found a decrease in proteins and/or lipids both in the mantle and the digestive gland of different species of cephalopods either sampled in the wild (Pollero and Iribarne, 1988; de Moreno et al., 1998; Zamora and Olivares, 2004; Kilada and Riad, 2008) or as observed from lab studies (Castro et al., 1992; García-Garrido et al., 2010; Morillo-Velarde et al., 2013).

In light of the morphometric and biochemical analyses, most authors have proposed an income energy model for reproduction in several cephalopod species such as ommastrephids (e.g., Arkhipkin and Silvanovich, 1997; Arkhipkin et al., 1998), loliginids (e.g., Hatfield et al., 1992; McGrath Steer and Jackson, 2004), sepiolids (e.g., Moltschaniwskyj and Carter, 2013) or octopods (e.g., Pollero and Iribarne, 1988; Rosa et al., 2004a, b; Quetglas et al., 2011). This model would be justified either by not having found trade-offs between tissues, that is, a transference of energy from tissue to tissue, or by having observed a positive correlation between somatic and reproductive condition during maturation. However, this is in contrast with the theory postulated by other authors consisting of the muscle (i.e., mantle) and digestive gland providing some energy for reproduction (i.e., capital breeding) (e.g., O’Dor and Wells, 1978; Seibel et al., 2000; Jackson et al., 2004; Lin et al., 2019; Pascual et al., 2020).

The common octopus (*Octopus vulgaris* Cuvier, 1797) is one of the most commercially important cephalopods worldwide (Pierce et al., 2010). As for most cephalopods, it has a short life cycle of less than 2 years, growing rapidly to maturity, spawning once, often seasonally, and it is an ecological opportunist with labile populations (Jereb et al., 2015). Moreover, females of this species are also characterized by an asynchronic oocyte development and a synchronic ovulation (Rocha et al., 2001; Sieiro et al., 2016), dying after breeding. Common to other cephalopods, and octopods in particular, *O. vulgaris* has a protein and amino acid metabolism (Houlihan et al., 1990), and it has been generally defined as an income breeder during sexual maturation with no transference of storage reserves from tissue to tissue (Rosa et al., 2004a; Otero et al., 2007; Lourenço et al., 2014). However, it is unknown if this pattern varies during spawning and brooding. Therefore, given that the biochemical changes along maturity and the patterns of energy allocation or trade-offs for reproduction are still poorly understood, we combined morphometric and detailed biochemical data throughout the entire reproductive cycle of female *O. vulgaris* to (i) study the biochemical changes along reproduction in different tissues accounting for other factors, (ii) compare the fatty acid composition between fully mature females and other wild life stages sourced from the literature, and (iii) determine the strategy for energy acquisition during the whole reproductive cycle of this species.

## Materials and Methods

### Sampling and Morphometric Measures

A total of 212 female *Octopus vulgaris* individuals from the creel fishery were obtained from landings at four ports in Galicia (NW Spain) from 2004 to 2007. Additionally, aiming to study all maturity stages, 34 females were obtained from experimental ongrowing in cages set up in mussel rafts (Chapela et al., 2006) from which 26 individuals were spawning or post-spawning females. All females (*n* = 246; 71 in winter, 65 in spring, 77 in summer and 33 in autumn) were used for subsequent morphometric and biochemical analyses (Supplementary Figure 1).

Maturation was assessed using a five-stage macroscopic maturity scale (I: immature, II: developing, III: maturing, IV: mature, V: spawning/post-spawning) proposed by Inejih (2000) and commonly used in the study region (Otero et al., 2007; Sieiro et al., 2014). Different morphometric measurements were also taken: body weight (BW), digestive gland weight (DGW), and ovary weight (OW), and two morphological indices were estimated: the gonadosomatic (GSI, GSI = OW/BW-OW) and digestive gland (DGI, DGI = DGW/BW-DGW) indices. Moreover, given that spawning and egg brooding are long-term processes (Garci et al., 2016), the 26 spawning and post-spawning females (i.e., macrostage V) were graded according to their ovary weights and the histological appearance of the ovaries. In doing so, we used a four-point scale, which ranged from 1, for females that had recently started laying the eggs and had large ovaries that showed some signs of atresia, to 4, for females that had already finished laying eggs and had highly atretic small ovaries (post-spawning) (Sieiro et al., 2016).

### Biochemical Analysis

The biochemical composition was analyzed in four different tissues from each female: arm, mantle, ovary and digestive gland. Samples from each tissue once taken were stored at −20°C until water content and lipids analyses, and at −80°C until analysis of protein and glycogen contents. Prior to study the biochemical composition, arm and mantle samples were skinned. All biochemical analyses were carried out in duplicate and the average was taken prior to subsequent statistical analysis.

Water content was determined by weight difference of the homogenized tissue (1–2 g) before and after 24 h at 105°C. The results were expressed as g water/100 g tissue. The lipid fraction was extracted by the Bligh and Dyer (1959) method, for which 10 and 15 g were employed of visceral (ovary and digestive gland) and muscle (arm and mantle) tissues, respectively. Lipid classes were assessed by different spectrophotometric methods: free fatty acid content was determined following Lowry and Tinsley (1976), phospholipids following Raheja et al. (1973), and sterols following Huang et al. (1961). Finally, triglycerides were first purified from the lipid extract by thin-layer chromatography and then measured according to Vioque and Holman (1962). Total proteins were determined following the Kjeldahl method AOAC (1990) with a Kjeltec 2300 analyzer unit (Foss Tecator, Barcelona, Spain). Furthermore, total carbohydrates (i.e., glycogen) were quantified as glucose according to Strickland and Parsons (1968). All lipid, protein and glycogen determinations were expressed in percentage of dry weight (DW).

To determine the fatty acid (FA) composition, total lipid extracts were converted into fatty acid methyl esters (FAME) according to the Lepage and Roy (1986) method. FAME were analyzed by GC/FID (Perkin-Elmer 8700 and Perkin Elmer Clarus 500 chromatographs). Peaks corresponding to FA were identified by comparison of their retention times with standard mixtures (Larodan, Qualmix Fish; Supelco, FAME Mix). Peak areas were automatically integrated with 19:0 FA which was used as an internal standard for quantitative purpose. The concentration of each FA or FA group was expressed as g/100 g of total FAME. The FA profile of the mature ovaries (i.e., ovaries in stage IV) was further compared to those values sourced from the literature for other life stages of the same species sampled in the wild (Supplementary Table 1). For this comparative analysis, we selected only those FA that were in common with the published studies.

### Energy Content

Energy density in each tissue was calculated by applying energy equivalents of 38.9, 23.6, and 16.7 kJ/g for lipid, protein and glycogen, respectively (Morillo-Velarde et al., 2013). In each tissue, total energy density (kJ/g) was calculated as the sum of the lipid, protein and glycogen energy densities. The energy (kJ) per tissue was determined by multiplying the energy density by the weight of each tissue for the ovary and the digestive gland. For arm and mantle, the gutted weight was considered and an average value of the arm and mantle energy densities was used to calculate the total energy for the whole muscle.

### Statistical Analyses

Differences in the mean concentration of each biochemical constituent among tissues were analyzed using a beta regression model with a logit link given that those variables were expressed as proportions. Additionally, the proportion of each constituent in each tissue was modeled as a function of maturity stage, season and tissue weight (BW for arm and muscle, OW for ovary and DGW for the digestive gland), also using beta regression models with a logit link as follows:

Y = α + β_1_X_1_ + β_2_X_2_ + β_3_X_3_, where Y would be the response variable, α would be the intercept, and β_n_ would represent the effects of X_n_ (i.e., maturity, season and tissue weight) on the response variable. Season and tissue weight were added to the formulation assuming to account for an influence on the biochemical changes related to time of the year and growth, respectively, not directly captured by the sexual maturation.

The relative importance of the different FA among tissues, maturity stages, and years, and the comparison of the FA profile in stage IV ovaries with published data on FAs for other life stages sampled in the wild (Supplementary Table 1) were analyzed using principal component analysis (PCA) based on the correlation matrix and normalized variables (i.e., subtracting the mean and dividing by the standard deviation). Bivariate relationships involving proportions as response variables were analyzed using beta regression models; otherwise, quantile regressions were used to account for heterogeneous relationships, and generalized additive models (GAM) to model non-linear relationships. All treatment of data and analyses were performed in R 3.6.1 (R Core Team, 2019) and using the ‘betareg 3.1-2’ (Cribari-Neto and Zeileis, 2010), ‘quantreg 5.52’ (Koenker, 2005), and ‘mgcv 1.8-31’ (Wood, 2006) libraries.

## Results

### Morphometric Changes With Maturity

**FIGURE 1:**
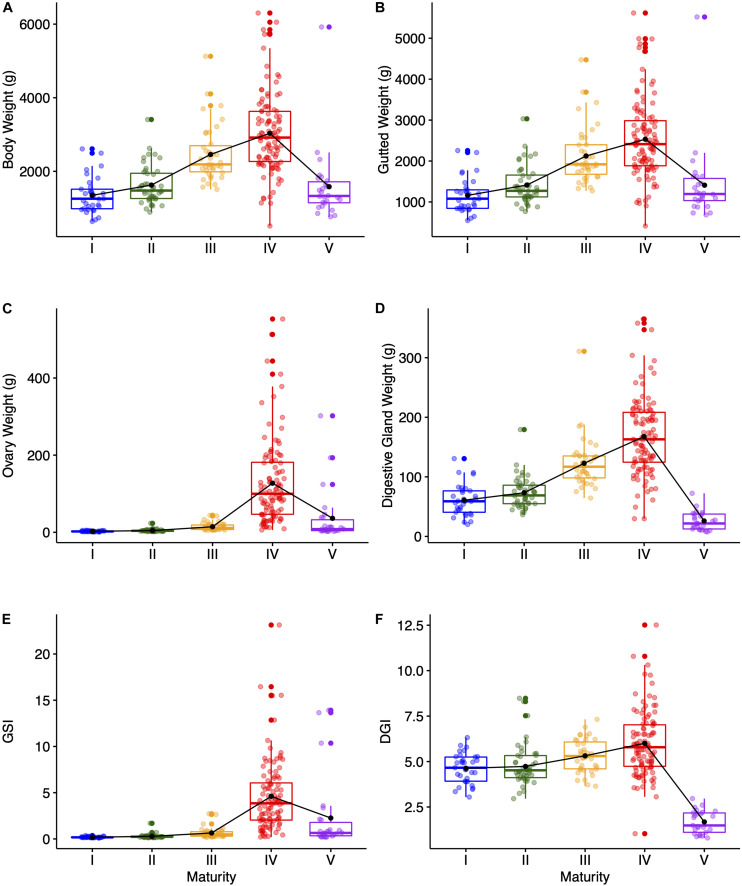
Boxplots showing the distribution of **(A)** body weight, **(B)** gutted weight, **(C)** ovary weight, and **(D)** digestive gland weight data across maturity stages. Shown are also the patterns in **(E)** GSI and **(F)** DGI.

The distribution of the morphometrical data across maturity macrostages is shown in Figure 1. Common to all morphometrics and indices, it was observed an increase from the immature stages to the fully mature stage (macrostage IV). However, while this increase was gradual throughout maturation for the somatic measurements (Figures 1A,B,D), the ovary weight (Figure 1C) and the GSI (Figure 1E) showed a sudden peak at macrostage IV. Moreover, this macrostage was characterized for showing a high variability in all morphometrics and indices, but mostly in the ovary weight and the GSI. Finally, it was observed a remarkable decrease in all morphometrics and indices at macrostage V reaching values of a similar magnitude as those for immature stages, with the exception of the digestive gland weight (Figure 1D) and notably the DGI (Figure 1F) that reached much lower values.

### Proximate Composition: Variability Among and Within Tissues

**FIGURE 2:**
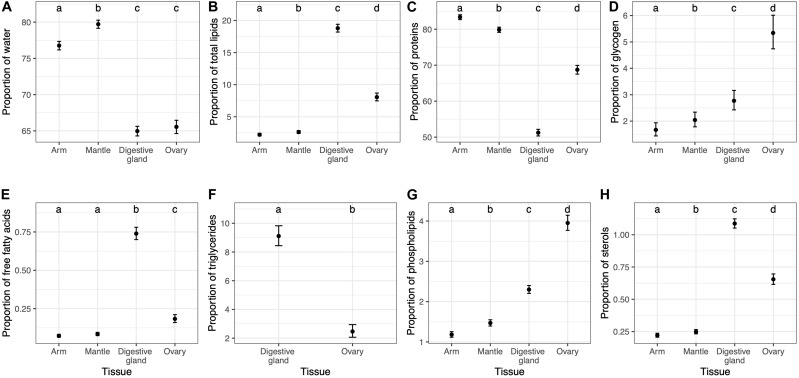
Average values (±95% C.I.) in % for water **(A)** and % of dry weight (DW) for the other biochemical constituents **(B–H)** measured in each of the four tissues (with the exception of triglycerides). Dots with shared letters on top indicate non-significant differences as evaluated with a beta regression model.

Water content was significantly higher in the muscle tissues (79.7 and 76.8% in mantle and arm, respectively) compared to the ovary (65.5%) and the digestive gland (64.9%) tissues (Figure 2A and Supplementary Table 2). Meanwhile, the highest lipid content was found in the digestive gland (19.8%), followed by the ovary (7.8%), and with the lowest values obtained in the muscle tissues (1.9 and 1.5% in mantle and arm, respectively) (Figure 2B and Supplementary Table 2). The different lipid classes (free fatty acids, triglycerides and sterols) followed the same pattern as the total lipid content, so that the highest values occurred in the digestive gland followed by the ovary and muscle tissues (Figures 2E,F,H and Supplementary Table 2). However, the ovary showed the highest value for phospholipids content (Figure 2G and Supplementary Table 2). Regarding total protein content, a significantly higher concentration was found in the muscle tissues (83.7 and 79.9% in arm and mantle, respectively) as compared to the ovary (68.9%) and the digestive gland (51.2%) tissues (Figure 2C and Supplementary Table 2). In the case of glycogen, the highest content was found in the ovary (5.4%), followed by the digestive gland and the mantle (2.6 and 2.2%, respectively), and with the lowest content occurring in the arm (1.6%) (Figure 2D and Supplementary Table 2).

**FIGURE 3:**
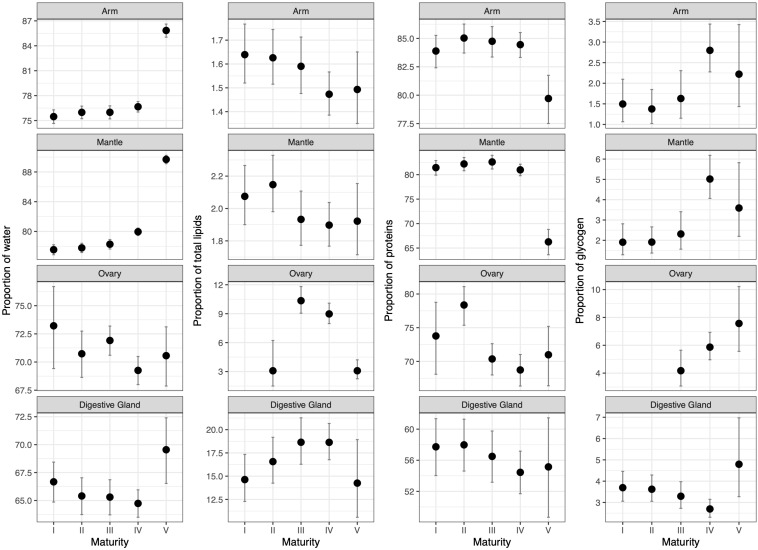
Differences in the proportion of each of the four main biochemical constituents (across columns) per tissue (along rows) during the reproductive cycle once accounting for seasonality (Supplementary Figure 3) and tissue weight (body for arm and mantle tissues, ovary or digestive gland log-transformed weight) (Supplementary Figure 5) as obtained from fitting beta regression models. Predicted values were obtained for an individual of 1.5 kg in spring.

Biochemical changes across maturity macrostages in each tissue and accounting for season and tissue weight are presented in Figure 3 and Supplementary Figures 2-6. Regarding the biochemical changes during sexual maturation, water content slightly increased and decreased in muscle and visceral tissues, respectively, from macrostage I to IV (Figure 3). Subsequently, all tissues showed a major increase in water content, particularly in the muscle and digestive gland, in spawning and post-spawning females (macrostage V). For total lipids, a slight downward trend was observed in muscle tissues from immature to mature/spawning females; whereas in visceral tissues, there was a significant increase up to macrostage III and IV for the ovary and digestive gland, respectively. Then, a subsequent decrease in both tissues for spawning and post-spawning females was observed. In both muscle tissues, the decrease in total lipids was mainly due to the changes in phospholipids while free fatty acids continuously increased during the reproductive cycle and sterols peaked in spawning and post-spawning females (Supplementary Figure 2). A very similar pattern to that of total lipids in the ovary was found for triglycerides, sterols and phospholipids (Supplementary Figure 2), with the main contribution given by the latter. Whereas, in the case of the digestive gland, the steady increase up to macrostage IV was mainly due to the content of triglycerides, while the decrease in macrostage V was caused by the rest of the lipids (Supplementary Figure 2). Concerning total protein content, this constituent remained practically constant in the arm and mantle during maturation, and dropped significantly in these tissues in spawning and post-spawning females, showing an opposite pattern to water content (Figure 3). In the ovary, total proteins decreased from immature to mature females, while in the digestive gland hardly any variation was found for this constituent along maturation (Figure 3). Finally, glycogen content in the muscle tissues showed an increase along maturation with a maximum value in macrostage IV (Figure 3). Similarly, glycogen in the ovary increased also from macrostage III to macrostage V. By contrast, this compound in the digestive gland showed a slight downward trend from immature to mature females to finally rise in the spawning and post-spawning stage (Figure 3).

Regarding seasonality, we found a few pronounced changes in the main four biochemical constituents. More specifically, in muscle tissues, arm and mantle, water and total lipid contents increased toward autumn, while proteins remained practically constant along the year (Supplementary Figure 3). This increment in the lipid content of the muscle was mainly due to sterols content (Supplementary Figure 4). In the ovary, there were not significant differences among seasons for any of the main biochemical components. The digestive gland showed the highest variability among seasons for total proteins and glycogen contents with opposite seasonal patterns between lipids and proteins. The variation found along seasons in the lipid content was mainly due to free fatty acids, with their values being higher in summer and lower in autumn (Supplementary Figure 4). Finally, the glycogen content was higher in spring in all tissues but only significantly higher than in summer in the case of the digestive gland (Supplementary Figure 3).

The relationship with tissue weight varied between biochemical constituents and tissues. More specifically, the proportion of water decreased with weight in all tissues (Supplementary Figure 5), total lipids decreased with the body weight in the arm (Supplementary Figure 5) due to free fatty acids and sterols (Supplementary Figure 6), but increased with digestive gland weight (Supplementary Figure 5) due to triglycerides (Supplementary Figure 6). Moreover, total protein content decreased with body weight and digestive gland weight, while it increased with ovary weight (Supplementary Figure 5). Finally, glycogen content increased with weight in visceral tissues, mainly with the digestive gland weight (Supplementary Figure 5).

**FIGURE 4:**
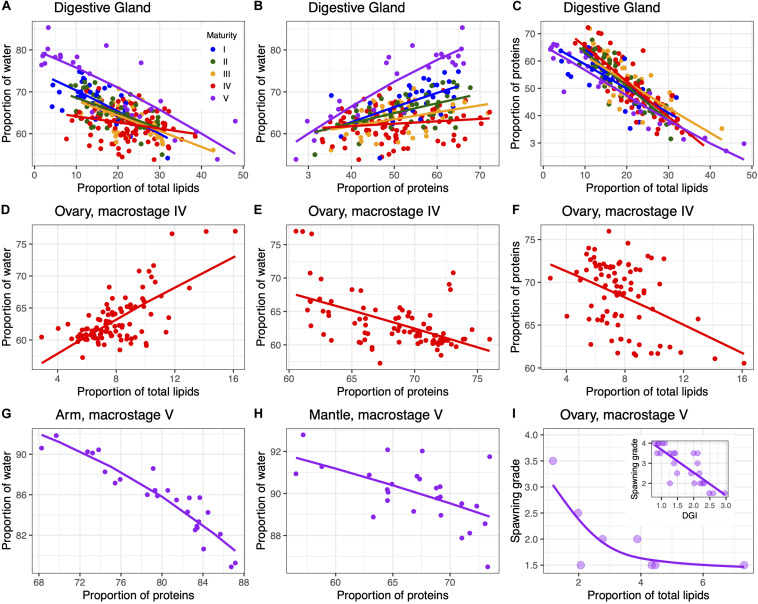
Scatterplots showing the relationships between the proportion of water, total lipids and total proteins for all maturity stages in the digestive gland **(A-C)**, in macrostage IV ovaries **(D-F)**, and macrostage V in the arm **(G)** and mantle **(H)**. Shown is also the relationship between total lipids (and condition index, DGI) with the spawning grade in macrostage V ovaries **(I)**. Lines show beta regression fits with the exception of panel **(I)** that shows GAM fits. See Supplementary Table 3 for numerical results.

Bivariate plots of the main biochemical constituents along maturation showed strong relationships mostly in the digestive gland and ovary (Figure 4 and Supplementary Table 3). More specifically, in the digestive gland the proportion of water was negatively correlated with the proportion of lipids (Figure 4A) and positively related to the proportion of proteins (Figure 4B) across all maturity stages. The strength of these relationships decreased with maturation, mainly in the latter case, though the steepest slope was found for post-spawning females in both cases. Consequently, the proportion of proteins and lipids were inversely related for all maturity stages in the digestive gland (Figure 4C). For the remaining tissues, some statistically significant relationships were observed for specific maturation stages. In particular, in mature ovaries, the relationship between water and lipid contents (Figure 4D) and water and protein contents (Figure 4E) showed an opposite pattern to the one observed in the digestive gland, while the proportion of proteins and lipids was again negatively correlated (Figure 4F). In muscle tissues, the negative relationship between water and proteins was only evident for spawning and post-spawning females (macrostage V) (Figures 4G,H). Finally, in macrostage V ovaries, females with a higher spawning grade had a much lower proportion of lipids and were in a poorer condition (Figure 4I).

### Fatty Acid Composition

**FIGURE 5:**
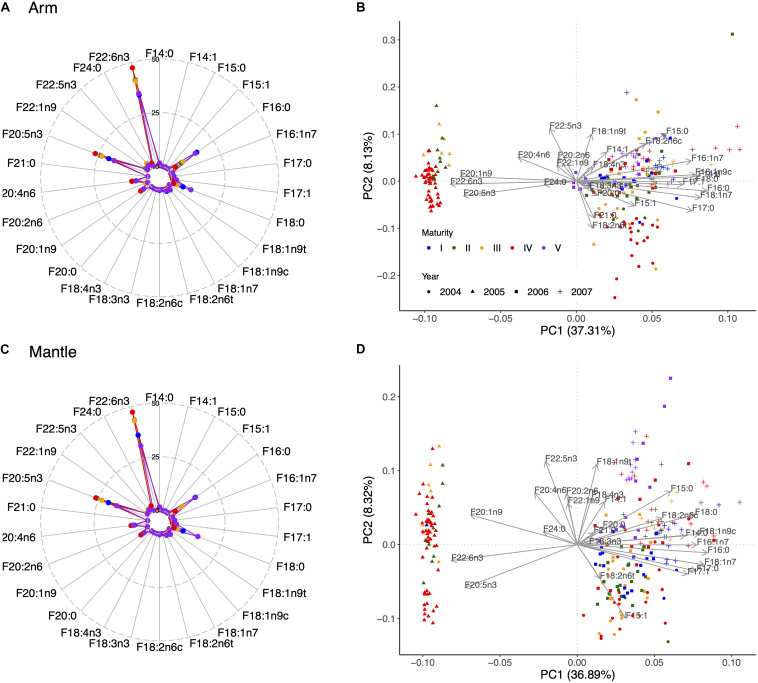
Radar plots showing the percentage of each of the analyzed fatty acids (FA) across maturity stages in the arm **(A)** and mantle **(C)**. **(B,D)** Illustrate the principal component analyses in the arm and mantle, respectively, showing the contribution of each analyzed FA across maturity stages and sampled years.

**FIGURE 6:**
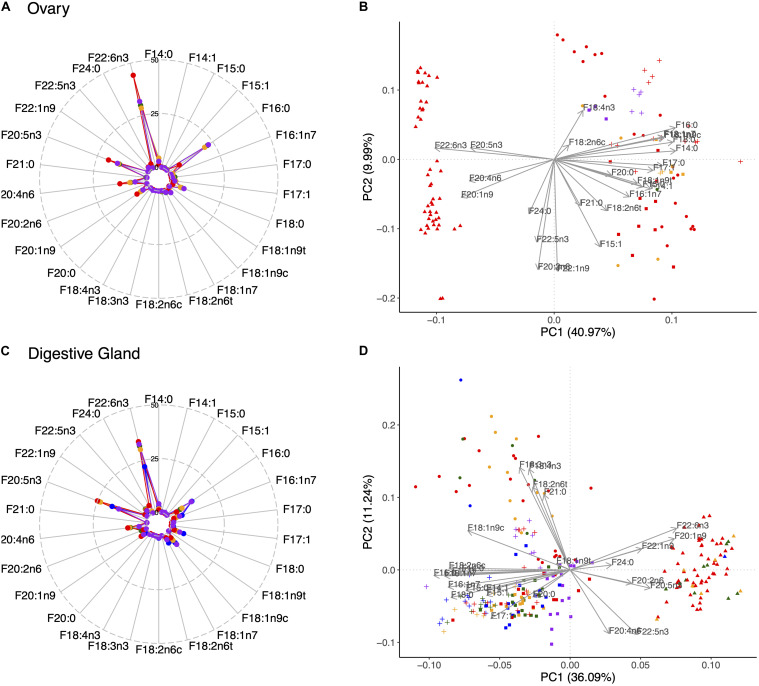
Radar plots showing the percentage of each of the analyzed fatty acids (FA) across maturity stages in the ovary **(A)** and digestive gland **(C)**. **(B,D)** Illustrate the principal component analyses in the ovary and digestive gland, respectively, showing the contribution of each analyzed FA across maturity stages and sampled years. Legend coding as in Figure 5.

The most abundant FA in all tissues were 16:0, 18:0, 20:1n9, 20:4n6, and especially 20:5n3 and 22:6n3 (Supplementary Table 4 and Figures 5, 6). The stearic (18:0), docosahexaenoic (22:6n3; DHA) and eicosapentaenoic (20:5n3; EPA) acids were more abundant in muscle tissues than in the viscera (compare Figures 5A,C with Figures 6A,C). Furthermore, the highest concentrations of palmitic (16:0), eicosenoic (20:1n9) and araquidonic (20:4n6; ARA) acids were particularly found in the ovary (Figure 6A). Certain monounsaturated FA (MUFA) such as 16:1n7, 18:1n7 and 18:1n9c (oleic acid) were also important in the digestive gland (Figure 6C). Interestingly, it could be observed that year 2005 was separated from the other years characterized by a high content of FA with 20 or more carbon atoms in all tissues (Figures 5B,D, 6B,D). A separation of year 2004 in the digestive gland was also noteworthy, and to a lesser extent in the arm as well, mainly due to 18:2n6t and 21:0. Finally, the content of those FA with 20 or more carbon atoms (20:1n9, 20:4n6, 20:5n3; and 22:6n3) was, on average, higher in mature females (macrostage IV) in all tissues (Figures 5B,D, 6B,D and Supplementary Figure 7), whereas immature (macrostage I) and/or spawning and post-spawning females (macrostage V) showed a higher content of FA with less than 20 carbon atoms (16:0, 16:1n7, 18:0, 18:1n7, and 18:1n9c) in all tissues as well (Figures 5B,D, 6B,D and Supplementary Figure 8).

**FIGURE 7:**
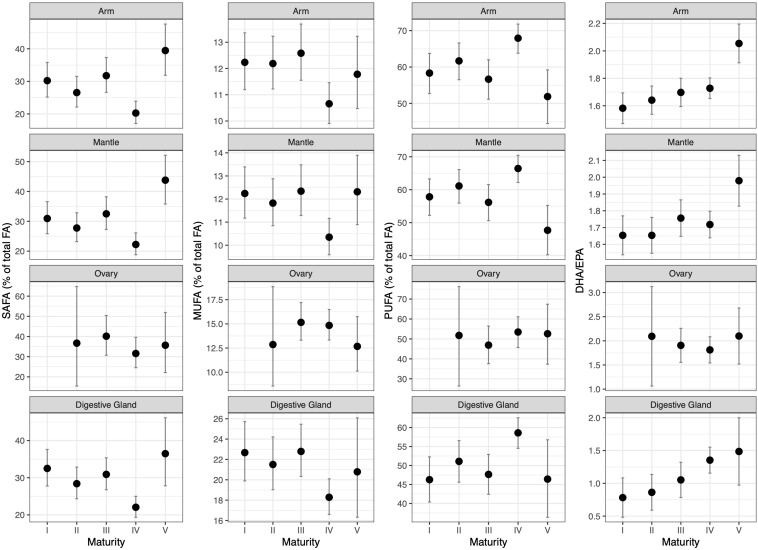
Differences in the proportion of fatty acids groups (SAFA, MUFA, and PUFA) and DHA/EPA ratio (across columns) per tissue (along rows) across maturity stages once accounting for seasonality (Supplementary Figure 9) and tissue weight (body for arm and mantle tissues, ovary or digestive gland log-transformed weight) (Supplementary Figure 10) as obtained from fitting beta regression models. Predicted values were obtained for an individual of 1.5 kg in spring.

Regarding FA groups, the content of saturated FA (SAFA) was higher in the muscle tissues (22.6%), followed by the ovary (22.4%) and the digestive gland (21.96%). The content of MUFA was significantly higher in the digestive gland (19.2%) as compared to the ovary (12%) and muscle tissues (10.9%). Remarkably, the polyunsaturated FA (PUFA) content was relevant in the muscle tissues (66.4% on average for arm and mantle) and the ovary (65.6), followed both by the digestive gland (58.8%) (Supplementary Table 4). The FA groups varied with maturity, season and growth. In particular, the lowest proportions of SAFA and MUFA were observed at macrostage IV, while SAFA increased in spawning and post-spawning females (macrostage V) especially for the muscle tissues and the digestive gland. The opposite pattern was evident for PUFA (Figure 7). The DHA/EPA ratio increased in the muscle and the digestive gland from immature to spawning and post-spawning females, and did not vary in the ovary (Figure 7). Regarding seasons, higher SAFA values were found in winter and spring than in summer and autumn in all tissues except for the ovary, while the opposite pattern was evident for PUFA (Supplementary Figure 9). The DHA/EPA ratio remained practically constant in all seasons for the muscle tissues and ovary, whereas in the digestive gland the highest and lowest values occurred in autumn and in spring, respectively (Supplementary Figure 9). Finally, the FA groups shared patterns in the relationship with tissue weight in which SAFA and MUFA contents decreased during growth, whereas the proportion of PUFA increased (Supplementary Figure 10). Interestingly, the DHA/EPA ratio decreased with body weight in both muscle tissues, while it increased with ovary weight and decreased with the digestive gland weight (Supplementary Figure 10).

### Fatty Acid Comparison Between Mature Ovaries and Other Life Stages

**FIGURE 8:**
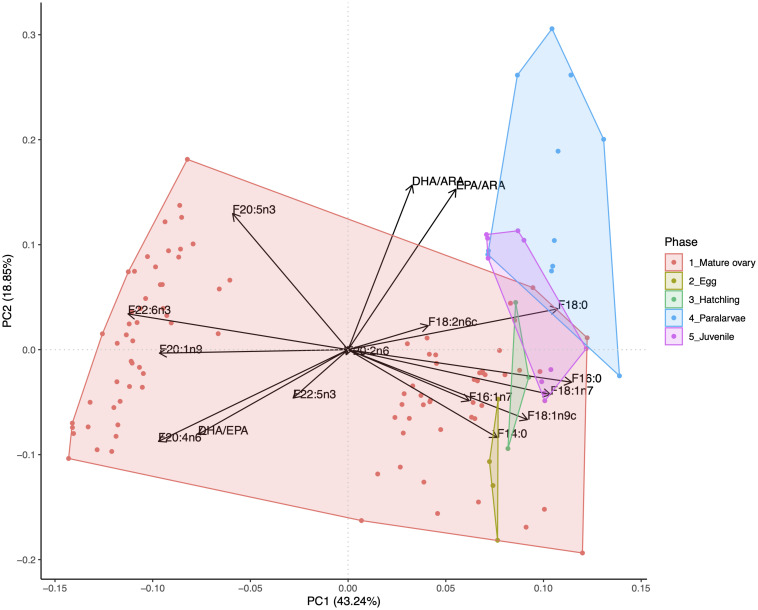
Principal component analysis showing the contribution of each analyzed fatty acid from mature ovaries studied in this work as compared to data sourced from the literature (Supplementary Table 1) of different wild life stages. See Supplementary Figure 11 for a PCA excluding the most extreme left values corresponding to ovaries from year 2005.

The FA profile in mature ovaries (macrostage IV females excluding those from the cages) was compared to different wild developmental stages of *O. vulgaris* (egg, hatchling, paralarva and juvenile) obtained from the literature (Supplementary Table 1). The PCA showed a separation of the different life stages with PC1 explaining 43.24% of the total variance and separating part of the mature ovaries from the remaining mature ovaries and the other developmental stages. This separation can be explained as a result of the high percentage of FA with 20 or more carbon atoms found in 2005 samples (Figure 8). PC2, which explained 18.85% of the total variance, separated the paralarvae from the rest of the developmental stages, with eggs, hatchlings and juveniles being more similar to the mature ovaries (Figure 8). The highest contribution to this separation was due to the higher DHA/ARA and EPA/ARA ratios in paralarvae. When excluding ovaries sampled in 2005, the grouping of life stages was more evident, though the influence of the FA profile in separating the groups was fairly similar (Supplementary Figure 11). More specifically, the separation of paralarvae from the other developmental stages was due to a higher content in those FA with less than 20 carbons in the paralarvae (mainly 16:0, 17:0, 18:0 and C18 unsaturated forms), whereas mature ovaries mostly showed higher contents of unsaturated FA with 20 or more carbon atoms such as ARA (20:4n6) (Supplementary Figure 12).

### Energy Content

**FIGURE 9:**
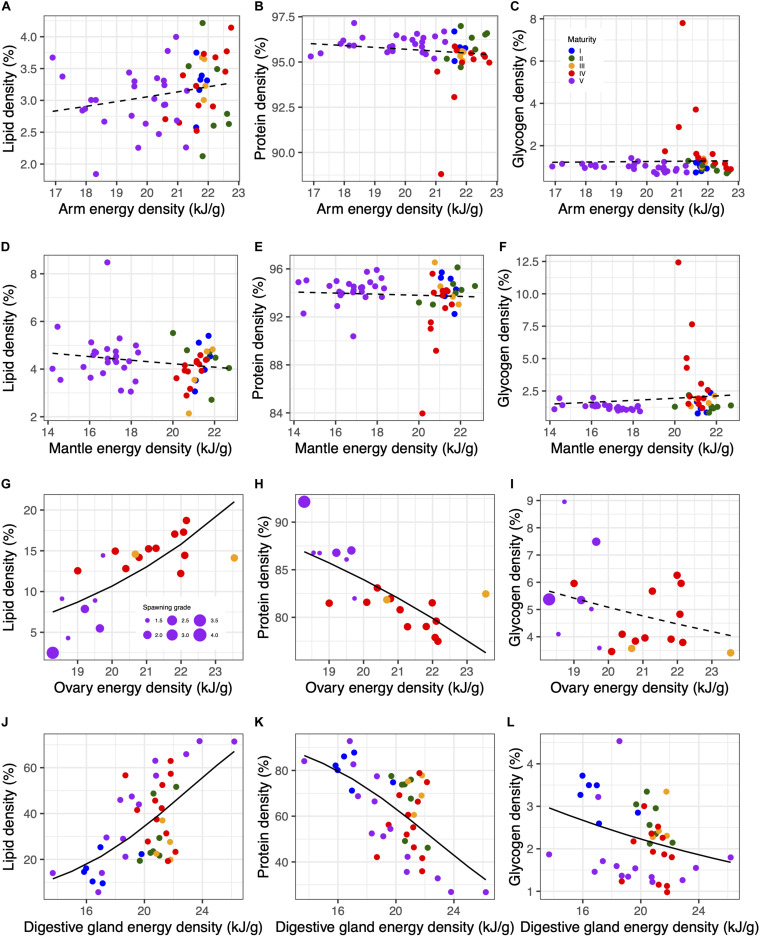
Relationships between constituent (lipid, protein and glycogen) density and energy in the arm **(A–C)**, mantle **(D–F)**, ovary **(G–I)** and digestive gland **(J–L)**. Dots are colored by maturity macrostage and spawning, and post-spawning individuals are scaled according to spawning grade. Lines show beta regression fits with solid lines indicating that the slope is statistically significant (*p* < 0.05). See Supplementary Figure 13 for a plot excluding glycogen from the density calculation. See Supplementary Table 3 for numerical results.

The variation in the energy density was mainly due to total lipids and proteins in visceral tissues, whereas muscle tissues showed little variation in their energy density (Figure 9 and Supplementary Table 3). On the one hand, there was hardly any variation of the energy density in the arm and mantle due to the contribution of total lipids (Figures 9A,D) or proteins (Figures 9B,E). On the other hand, both visceral tissues showed a similar variation of the energy density supplied by total lipids and proteins, reaching maximum values of ∼20% and >60% for total lipids (Figures 9G,J), and minimum values of ∼80% and <40% for total proteins (Figures 9H,K), in the ovary and the digestive gland respectively. Thus, higher energy densities in the ovary and digestive gland were found when the contribution of total lipids increased and that of proteins decreased. Finally, glycogen content showed the lowest contribution to energy densities in all tissues, becoming more important in the ovaries of spawning and post-spawning females (macrostage V) (Figures 9C,F,I,L). Removing this constituent from the calculation of energy densities (note the reduced number of samples where glycogen was measured; Supplementary Table 2) hardly changed the patterns described above (Supplementary Figure 13).

**FIGURE 10:**
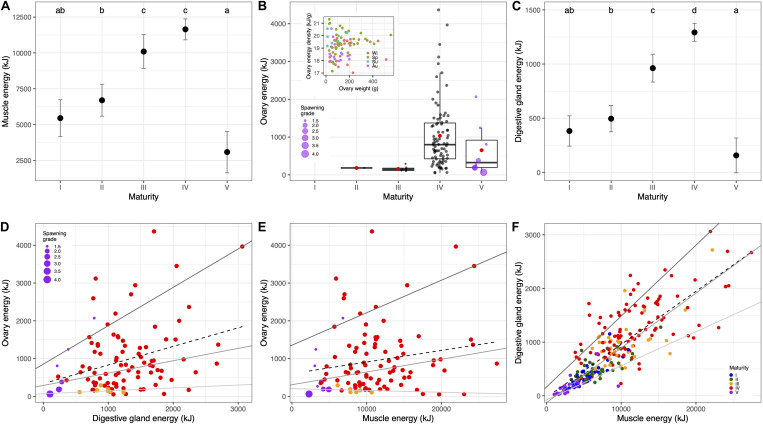
Changes in energy across maturity stages in the muscle **(A)**, ovary **(B)**, and digestive gland **(C)**. The inset in **(B)** shows a scatterplot of the ovary weight and ovary energy density colored by season for macrostage IV females. **(D–F)** Show the relationships between energy content in specific tissues illustrating the energetic trade-offs. Dots are colored by maturity macrostage, and spawning and post-spawning individuals are scaled according to the spawning grade in **(D,E)**. Lines in **(D–F)** show quantile regression fits for 0.90, 0.50, 0.10 quantiles (solid lines from dark gray to light gray), and least squares regression (dashed line). See Supplementary Table 3 for numerical results, and Supplementary Figure 14 for a plot including glycogen in the energy content calculation.

The variation of total energy across maturity stages for each tissue is shown in Figure 10 without the contribution of glycogen since its input in the energy density was very low in all tissues as described above (see however Supplementary Figure 14 for comparison). On the one hand, there was a clear pattern related to reproduction, since a maximum was reached at macrostage IV. This pattern was very similar for muscle (Figure 10A) and digestive gland (Figure 10C), with a significant steady increase until a maximum was reached at macrostage IV, and a subsequent drop reaching a minimum of energy at spawning and post-spawning females (macrostage V). In the ovary, there was also a maximum at macrostage IV, and a consecutive loss of energy at macrostage V which varied across individuals depending on the spawning grade (Figure 10B). Moreover, at macrostage IV, the energy in the ovary was very variable with the highest variation in energy density occurring at ovary weights roughly below 200 g in all seasons, whereas energy density was more stable for ovaries larger than 200 g in winter and spring (see inset in Figure 10B). On the other hand, the energy relationships between somatic and reproductive tissues, that is trade-offs, showed that these were positive and slightly heterogeneous in the case of the energy storage in the digestive gland and the muscle (Figure 10F and Supplementary Table 3). However, this relationship was also positive though highly heterogeneous between the ovary and digestive gland (Figure 10D and Supplementary Table 3), and mostly between the ovary and muscle (Figure 10E and Supplementary Table 3) resulting in a non-significant relationship in this latter case.

**FIGURE 11:**
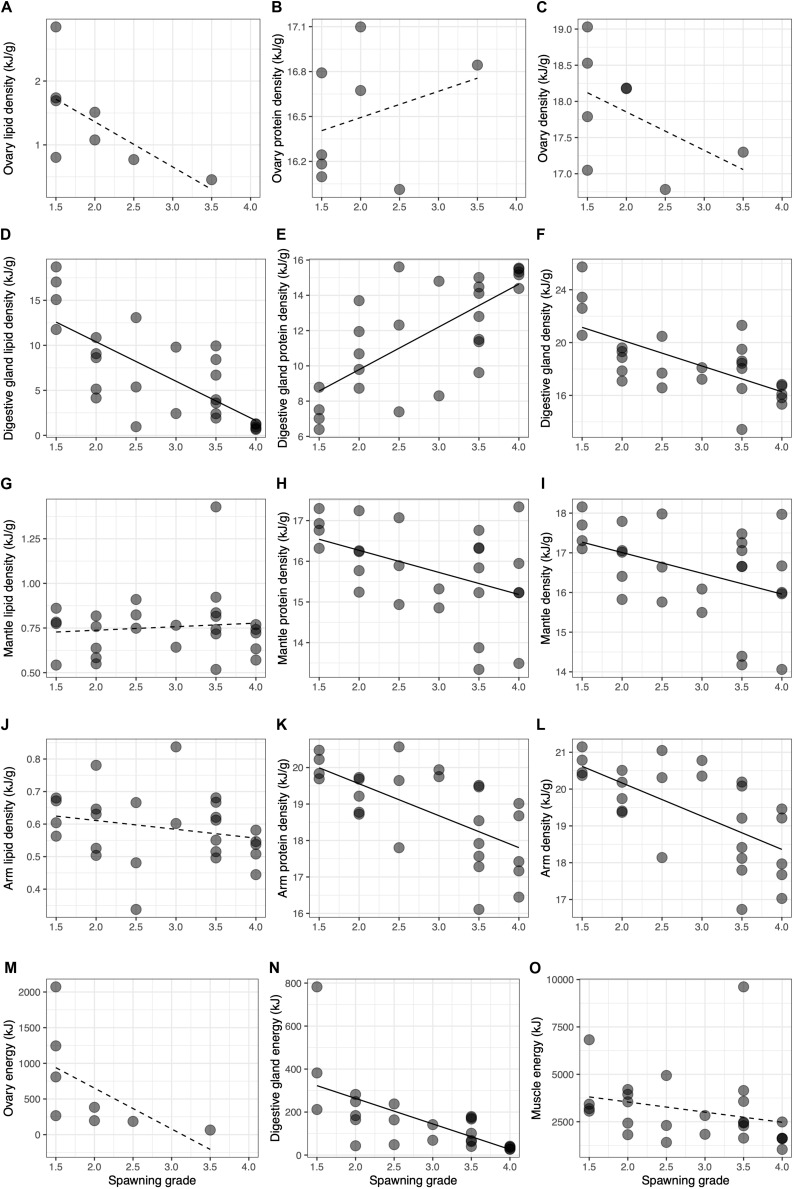
Bivariate relationships between energy density and spawning grade for lipids **(A,D,G,J)** and proteins **(B,E,H,K)** for each tissue in macrostage V females. Shown are also the relationships between energy density **(C,F,I,L)** and total energy **(M,N,O)** for each tissue. Solid lines indicate that the slope is statistically significant (*p* < 0.05).

Finally, the specific variation in the energy density and total energy (excluding the contribution of glycogen) as a function of the spawning grade for macrostage V females is shown in Figure 11. In all tissues, there was a generalized decrease both in the energy density (Figures 11C,F,I,L) and total energy (Figures 11M–O) during the breeding period. The loss of energy density was due to a decrease in the total lipid content in visceral tissues, though only significant in the digestive gland (Figures 11A,D), whereas muscle tissues showed a reduction in the total protein content (Figures 11H,K). In terms of total energy, visceral tissues (Figures 11M,N) showed a higher decrease compared to the muscle tissue during the breeding period (Figure 11O), though it was only significant in the digestive gland.

## Discussion

### Biochemical Composition During the Reproductive Cycle

#### Metabolism and Proximate Composition

Based on the proximate composition and energy density in the muscle and visceral tissues throughout reproduction, *O. vulgaris* has a protein-based metabolism with proteins as the major organic material, and energy reserves primarily located in the body muscle as previously observed by others (e.g., Houlihan et al., 1990; Rosa et al., 2002). All tissues’ energy densities were determined by the protein content throughout reproduction as commonly happens in cephalopods (Lin et al., 2017, 2019) and other mollusks such as bivalves (Vite-García and Saucedo, 2008). In fact, the highest dependence on this energy resource occurred in the muscle tissues (arms and mantle), whereas the visceral tissues (ovary and digestive gland) also depended on lipids and glycogen. More specifically, the contribution of glycogen was more relevant in the ovary agreeing with previous research on this species and other octopods (Rosa et al., 2004a, b). However, our values of glycogen content in the digestive gland and the muscles of female *O. vulgaris* were remarkably lower than those values obtained by Rosa et al. (2004b). In addition, lipids were even more important than proteins in the digestive gland for advanced maturity stages. In fact, the different lipid classes also predominated in the digestive gland, with the exception of phospholipids that were highest in the ovary agreeing with previous findings in this species and other cephalopods (de Moreno et al., 1998; Sieiro et al., 2006).

Maturity, unlike seasonality and growth that in general played a minor role, was the main factor determining the proximate composition of female *O. vulgaris* as observed for other cephalopods (Kilada and Riad, 2008). In this sense, regarding the biochemical changes along maturation, we found both similarities and differences with other studies. The major changes from an immature to a mature stage in muscle tissues were due to an increase in the water and glycogen contents, while total lipids and proteins remained practically unaffected. This is in contrast with the decrease in the glycogen content found by Rosa et al. (2004b) in the mantle of the same species. Contrary to muscle tissues, the digestive gland in mature females showed a decrease in the water and glycogen contents together with an increase in the total lipid content mainly due to triglycerides. The decrease in the glycogen content was previously observed in the same species by Rosa et al. (2004b) as well as the increment in the total lipid content (Rosa et al., 2004a; Lourenço et al., 2014). In the case of the ovary, the main changes occurred by a decrease in water and total protein contents together with an increase in glycogen and total lipids. This increase in glycogen agrees with results obtained by Rosa et al. (2004b) for *O. vulgaris* and by Olivares et al. (2019) for *Octopus mimus* suggesting that glycogen would be important during sexual maturation and embryogenesis. Regarding total proteins and lipids, the former reached a maximum in developing ovaries (macrostage II), while lipids, in particular phospholipids and sterols, peaked during the maturing stage (III). In this sense, Buckley (1977) and Boyle and Chevis (1992) pointed out that the accumulation of lipids in the ovary occurs until the onset of vitellogenesis and, subsequently, the accumulation of glycoproteins begins. However, taking our results together, we suggest that there could be a different succession of yolk-forming molecules, more specifically, proteins would mainly contribute to immature ovaries at first, then lipids in maturing ovaries, and lastly, glycogen in mature ones. Taking into account that the ovary is made up mostly of proteins, this could respond to the main need of forming cell structures such as membranes and follicle cells throughout the developing phase. As the ovary matures, there would be a higher incorporation of lipids followed by glycogen to form the yolk as observed for the squid *Lolliguncula panamensis* (Arizmendi-Rodríguez et al., 2012). In line with this, recent studies have observed an increment in the transcript abundance of two vitellogenins that are glycolipoproteins in the ovarian follicle cells during the late vitellogenic phase of two squids (Kitano et al., 2017; Chen et al., 2018).

Biochemical characterization of spawning and post-spawning stages has been hardly studied in cephalopods and octopods in particular. We incorporated those stages into our study from individuals ongrown in suspended cages due to the difficulty of getting them directly from the wild given the strategy of hiding for taking care of the eggs. We acknowledge that this fact may have an influence on the biochemical basis due to the differences in diet between the cages and the wild; however, we believe that the overall trends (increase or decrease) of the compounds would not be altered. That being said, at macrostage V and throughout breeding, represented by the spawning grade, the major changes at the biochemical level were the decrease in total proteins in the muscle tissues and the decrease in total lipids in the visceral tissues. The loss of proteins in the muscle tissues was previously discussed for the female and male of the same species (Rosa et al., 2002) and also found in other cephalopods such as *Octopus tehuelchus* (Pollero and Iribarne, 1988), *Sepia officinalis* (Castro et al., 1992), and *Loligo forbesi* (Kilada and Riad, 2008). In relation to the digestive gland, García-Garrido et al. (2010) also found a decrease in lipids for males of the same species, specifically in triglycerides, suggesting a mobilization of this lipid class from this organ to muscular tissues during short starvation (Morillo-Velarde et al., 2013). Other authors also described the consumption of SAFA and MUFA throughout fasting (Castro et al., 1992; García-Garrido et al., 2010). Similarly, the ovary showed a decrease in total lipids probably due to egg expelling during spawning, since lipids are an inherent constituent of the egg yolk. These results concur with previous observations in the ovary of spawning and post-spawning individuals of *O. tehuelchus* (Pollero and Iribarne, 1988). Furthermore, during breeding, the increase in the total protein content, represented by the energy density, in visceral tissues could be due to the synthesis and deposit of collagen fibers (fibrosis) as a typical symptom of dysfunction during senescence, as previously shown in *O. mimus* (Zamora and Olivares, 2004).

#### Fatty Acid Composition

Despite the low lipid concentration in octopus’s body, lipids are the limiting component for growth and egg production (O’Dor et al., 1984; Navarro and Villanueva, 2000, 2003). In fact, fatty acids are defined as important dietary constituents in cephalopods (Meynier et al., 2008; Lourenço et al., 2017). In this regard, we found considerable quantities of long-chain PUFA such as DHA and EPA in all tissues as well as other fatty acids such as 16:0, 20:1n9 and ARA mainly in the ovary, 18:0 mostly in the muscle tissues, and some monounsaturated fatty acids (16:1 and 18:1) mainly in the digestive gland as previously observed for this species (Sieiro et al., 2006). Moreover, the content in PUFA predominated over SAFA, and the latter over MUFA for all tissues similar to other cephalopods (de Moreno et al., 1998; Lin et al., 2019). Among tissues, the muscle (arm and mantle) was richer in PUFA and the digestive gland contained a significantly greater concentration of MUFA confirming earlier observations (Sieiro et al., 2006).

There was a remarkable opposite pattern between PUFA and SAFA in their relationships with maturity, season and growth. Regarding maturation, mature females showed higher PUFA content in the muscle tissues and the digestive gland, as it was previously found in the mantle of the same species (Rosa et al., 2002). However, the ovary did not show any significant changes in FA groups or in the DHA/EPA ratio with maturation. Other studies have observed an invariable DHA/EPA ratio in the ovary for the same species suggesting that this organ has a certain independence from food or trophic habitat along development unlike the other tissues (Lourenço et al., 2014). Moreover, Jackson (2005) also found a stable DHA/EPA ratio between seasons in *Todarodes filippovae*, and pointed out that the FA profile in the ovary and the ovulated eggs in the oviduct was relatively maintained between seasons in order to safeguard the nutritional quality of the eggs.

Regarding seasonality, the increment in the PUFA content during summer and autumn and a higher DHA/EPA ratio in autumn with both patterns mostly observed in the digestive gland, could be explained by changes in the diet that would be lately detected in the FA profile as shown in other cases (e.g., Hardy and Keay, 1972; Tir et al., 2015; Fuiman, 2018). Apart from the season, we observed other time-related variations associated to the sampled year, particularly in 2004 and 2005, when differences were mainly apparent in the FA profile of the digestive gland. Overall, changes in the FA profile in the digestive gland along time would probably indicate that this organ reflects nutritional changes in the animal’s preys and, alongside specific lipid classes, it would be a good indicator of differences in the dietary variations or trophic habitat between populations as previously suggested by others (Phillips et al., 2003; Fluckiger et al., 2008; Lourenço et al., 2014).

### Comparison of FA Profile Among Life Stages

The PCA carried out on the FA profile of the wild developmental stages of *O. vulgaris* revealed a clear separation of the paralarvae from the other stages due to a higher contribution of DHA/ARA and EPA/ARA ratios. Previous research showed a reduction in some SAFA (14:0 and 16:0) and ARA content from wild eggs to wild hatchlings, suggesting their use during the embryonic phase (Estefanell et al., 2017). In this regard, we observed a lower content in ARA in the paralarvae, which would explain the higher DHA/ARA and EPA/ARA ratios, together with a reduction in 14:0 content. Moreover, Estefanell et al. (2017) reported also higher contents of 18:1n9 and EPA in wild hatchlings compared to wild eggs suggesting their importance as phospholipids or energy substrate at this stage. We also detected an increment of 18:1n9 in the paralarvae, along with a higher content of some SAFA (e.g., 16:0) and other C18 unsaturated FA (e.g., 18:2n6c), which could be due to exogenous feeding as suggested by Lourenço et al. (2017).

According to our results, there was a high initial content of ARA in the developmental stages preceding the paralarval phase. This suggests that there would be a consumption of ARA up to the planktonic stages, accompanied by a retention or even an increase of EPA in the paralarvae. Later supply of ARA needed by the paralarvae could be sourced from food, in fact, Roura et al. (2012) pointed out that just a reduced number of species of decapods constitutes the natural preys at the early paralarval stage. These decapods are indeed richer in the content of ARA as compared to other preys (Quintana et al., 2015; Roo et al., 2017). Such a high catabolism of ARA in the paralarvae could be due to its important role throughout *O. vulgaris* development, since this FA is a substrate for biosynthesis of essential substances, such as prostaglandins (Agnisola et al., 1994), with several functions in marine species such as osmoregulation and reproduction (Mustafa and Srivastava, 1989; Hu et al., 2009). Moreover, ARA was recently found as a precursor for the biosynthesis of EPA in this species (Garrido et al., 2019), which could explain why EPA is the only long-chain PUFA that is maintained in a higher concentration in the paralarvae as compared to the mature ovaries.

### Allocation of Energy and Trade-Offs During the Reproductive Cycle

#### Sexual Maturation

During sexual maturation (i.e., transition from macrostages I to IV), the energy density in muscle tissues was quite constant due to the stable concentration of total proteins agreeing with previous observations for this species (Rosa et al., 2004a) and also for female *Illex argentinus* (Lin et al., 2017). Consequently, the increase in the total energy observed in muscle tissues from immature to mature females should be due to the organ’s growth. In fact, Houlihan et al. (1990) pointed out that rapid growth in *O. vulgaris* is brought about by high rates of protein synthesis as well as little protein degradation. Regarding visceral tissues, however, there was a higher variability in the energy density along sexual maturation with mature females showing greater energy densities than immature individuals mainly due to protein and lipid contents in both tissues. This was especially apparent in the digestive gland, contrasting with Rosa et al. (2004b) who did not find any significant energy density variation in this organ as well as in the muscle across sexual maturation probably due to the differences in the number of sampled females and/or the level of maturity staging. Regarding the ovary, we would expect a similar variability of energy density across maturity stages. However, unfortunately, immature ovaries (macrostages I and II) could not be analyzed for energetic purposes in our study. Nevertheless, such variability could be attributed to the vitellogenesis which is an intrinsic process during sexual maturation (Sieiro et al., 2014). This increment in the ovary energy density from immature to mature individuals associated to yolk accumulation was previously found for this species by Rosa et al. (2004b) as well as for other cephalopods (Lin et al., 2017), so as for other taxa such as fishes (Fernández et al., 2009). Therefore, the increase in the total energy in visceral tissues throughout sexual maturation could be due to, not only the organ’s growth as revealed by the increase in condition and gonadosomatic indices observed in previous works as well (Otero et al., 2007; Sieiro et al., 2014), but also to the accumulation of lipids mostly triglycerides in the digestive gland and phospholipids in the ovary. Finally, glycogen seems to have a minor contribution to the energy density in all tissues with the exception of the ovary where the contribution was higher. This latter relevance could be explained by the role of glycogen in the storage and mobilization of nutrients for ovary maturation, as suggested by Olivares et al. (2019) for *O. mimus* and Pascual et al. (2020) for *O. maya*, or because it is used later during embryogenesis as a source of energy as suggested by Rosa et al. (2004b) for this species.

All tissues accumulated energy along sexual maturation without negative energy relationships between somatic and reproductive tissues. Therefore, there was no apparent soma deterioration in pursuit of ovarian growth, in other words, we found no evidences of energy trade-offs between muscle or digestive gland to the ovary. However, while the energy in somatic tissues (i.e., muscle and digestive gland) increased gradually, the total energy in the ovary significantly peaked in the later maturity stage (IV). Most animals must attain a certain level of condition related to growth and stored products before maturation can occur (Moltschaniwskyj and Johnston, 2006). In fact, Otero et al. (2007) found that condition mainly increased between immature (II) and maturing (III) stages just prior to the highest gonadal investment (from III onward). Moreover, the relationships between somatic (body and digestive gland weights) and reproductive growth (ovary weight) showed a negative allometry until macrostage III. In the present work, the DGI steadily increased from macrostage II to IV, whereas the GSI did it from III to IV. This asynchrony was confirmed in energetic terms given that the greatest change in the total energy content occurred earlier for the muscle and digestive gland (between II and III) than in the ovary (between III and IV). Therefore, some reallocation of energy occurred from somatic growth toward gonad enlargement at maturing females (macrostage III). Furthermore, the ovary showed a high individual variability in the total energy content once the female reached maturity (macrostage IV). The main factor for this variability could be the difference in the ovary weights among seasons more than the variation in the energy density itself, with small ovaries (<200 g) observed throughout the year and large ovaries (>200 g) observed only in winter and spring. It is noteworthy that ovary weights in summer were smaller but highly energetic in terms of density. The existence of a higher variability in the ovary weights at macrostage IV and not in earlier stages, could be due to a high variability in fecundity and/or oocyte size that, in turn, would depend on female size (Otero et al., 2007). Therefore, the ovary weight would be restricted to the amount of body energy accumulated, which would be decisive during breeding. In teleost fishes, it has been shown a dependency between egg production and female size and energetics (e.g., Serrat et al., 2019). The mechanism by which ovary growth would be linked to body energy would be the ovarian atresia. Indeed, a massive atretic phenomena is produced in any late vitellogenic ovary (macrostage IV) suppressing all previtellogenic oocytes prior to spawning (Sieiro et al., 2016).

Taking into account our results, we might conclude that female *O. vulgaris* would be characterized by the use of an income strategy for energy acquisition during maturation. During sexual maturity, both somatic and reproductive growth would occur simultaneously, though reallocating part of the energy from the somatic growth to ovary growth once vitellogenesis starts (macrostage III, Sieiro et al., 2014) but without any deterioration of the soma, i.e., trade-offs, as happens in other cephalopods (e.g., Quetglas et al., 2011; Moltschaniwskyj and Carter, 2013). Therefore, the energy for ovary growth would come directly from food agreeing with previous findings for the same species (Rosa et al., 2004a; Otero et al., 2007) and other cephalopods (Moltschaniwskyj and Semmens, 2000; Rosa et al., 2005a).

#### Breeding

At macrostage V, females showed a decrease in the energy density in all tissues along with a concomitant loss in the total energy content, also highly evident in the decrease of reproductive and mainly somatic condition. On the one hand, the digestive gland showed a broader range of energy density for this stage compared to the ovary and muscle tissues suggesting that the digestive gland would be the first organ that experiences a change in weight. This observation in the wild would concur with results obtained during experimental starvation in several other cephalopod species for which an early loss in weight was first observed in this organ (Castro et al., 1992; García-Garrido et al., 2010; Roumbedakis et al., 2018). On the other hand, the decrease in the total energy content from mature to spawning and post-spawning ovaries occurred more progressively as revealed by the spawning grade, possibly due to the gradual egg expelling.

During breeding, represented by the spawning grade, we observed a decrease in the energy density and total energy in all tissues. This decrease was mainly apparent shortly after the onset of egg laying (i.e., spawning grade ≤ 2) concurring with observations made by Roumbedakis et al. (2018) who pointed out that the major changes in the physiological and immunological condition of brooding *O. maya* occurred immediately after spawning and before 10 days had elapsed. Furthermore, the absence of feeding during the long lasting period of breeding in this species (Wodinsky, 1978), which may be as long as 4 months in these waters (Garci et al., 2016), implies that spawning, maternal care and subsequent hatching should be financed by the energy accumulated during sexual maturation. In this sense, Roumbedakis et al. (2018) suggested that the ovary and digestive gland of *O. maya* were the main sources of lipids and the muscle the main source of proteins during maternal care. However, we propose that, on the one hand, the atretic process in the ovary would allow the female to recover part of the energy from those oocytes not finally laid, and thus, the spawning success would depend solely on the performance of the ovary. On the other hand, muscle tissues and the digestive gland, would supply the energy needed during maternal care in the form of proteins and lipids, respectively, for the body maintenance or homeostasis safeguarding the female survival until the last egg hatches.

## Data Availability Statement

The datasets generated for this study are available on request to the corresponding author.

## Ethics Statement

Ethical review and approval was not required for the animal study because the authors declare that research was done with individuals obtained from the commercial fishery that were captured according to the legal standards established in our region.

## Author Contributions

PS contributed with the conception of the study, the data acquisition, analysis, interpretation, and manuscript writing. JO contributed with the analysis, interpretation, and critical revision of the manuscript. SA contributed with the interpretation of the results and critical revision of the manuscript.

## Conflict of Interest

The authors declare that the research was conducted in the absence of any commercial or financial relationships that could be construed as a potential conflict of interest.
