# Supplementary material for: Biochemical Composition and Energy Strategy Along the Reproductive Cycle of Female Octopus vulgaris in Galician Waters (NW Spain)
Source: Front Physiol. 2020 Jul 15;11:760. doi: 10.3389/fphys.2020.00760 (PMC7373806; doi:10.3389/fphys.2020.00760)
Supplement: Supplementary file 1 [file Data_Sheet_1.PDF]

SUPPLEMENTARY MATERIAL

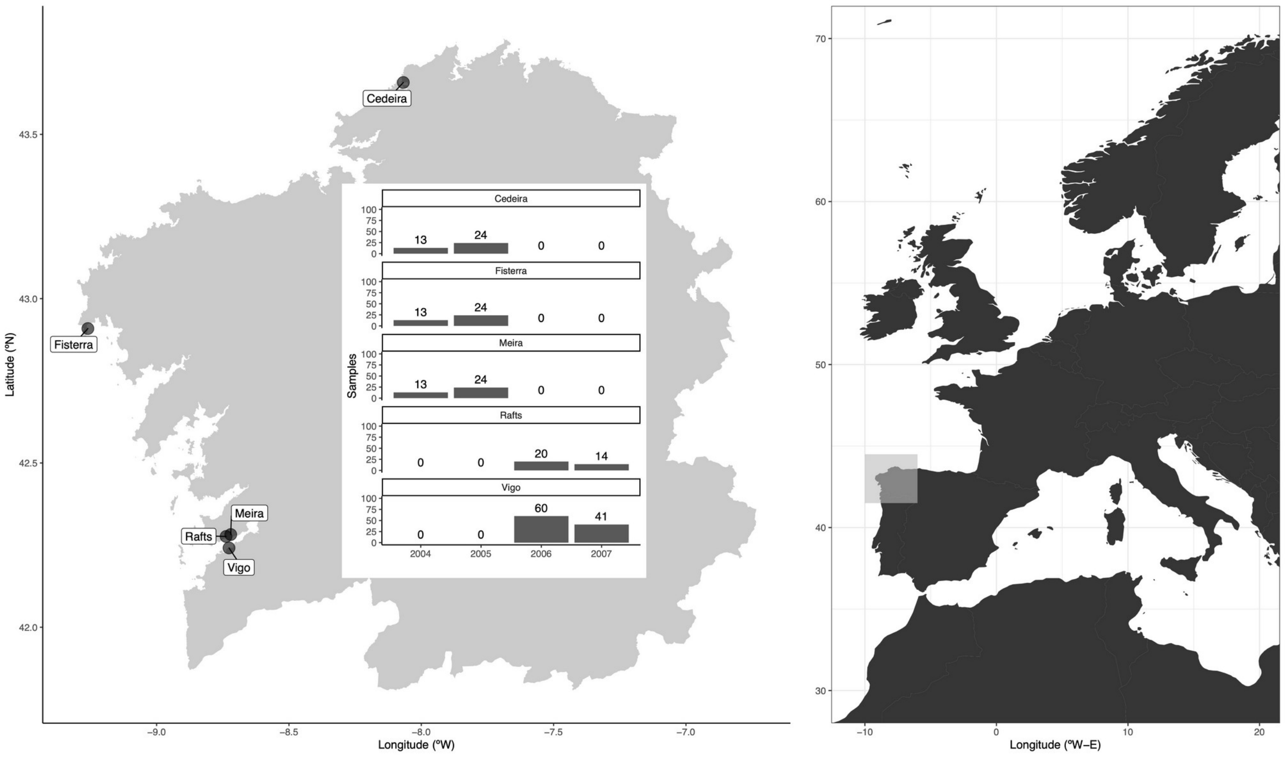

Supplementary Figure 1. Map showing the area of study and the location of the ports and ongrowing cages set up in mussel rafts where samples were obtained. The inset shows the number of individuals sampled in each port and ongrowing rafts per year.

Supplementary Table 1. List of studies used to compare the fatty acid profile in mature (macrostage IV) ovaries from the current study with those values sourced from the literature for other life stages of the same species sampled in the wild. Shown is also the number of samples (N) available from each study and life stage combination. Hatchling life stage is defined as newly hatched individuals, that is, individuals that did not feed on wild preys; whereas paralarvae are individuals that have been feeding in the wild during a certain period of time.

**Source**	**Life stage**	**N**	**Sampling location**
Navarro and Villanueva (2000)	Hatchling	1	Barcelona (NW Mediterranean Sea)
Navarro and Villanueva (2003)	Ovary, Egg, Juvenile	1, 3, 10	Barcelona (NW Mediterranean Sea)
Garrido et al. (2016)	Paralarvae	10	Ría de Vigo (NE Atlantic Ocean)
Estefanell et al. (2017)	Egg, Hatchling	1, 1	Canary Islands (CE Atlantic Ocean)
Lourenço et al. (2017)	Hatchling, Paralarvae	1, 1	Ría de Vigo (NE Atlantic Ocean)
This study	Mature ovary	90	Galicia (NE Atlantic Ocean)

References

Estefanell, J., Mesa-Rodríguez, A., Ramírez, B., La Barbera, A., Socorro, J., Hernández-Cruz, C. M., and Izquierdo, M. S. (2017). Fatty acid profile of neutral and polar lipid fraction of wild eggs and hatchlings from wild and captive reared broodstock of *Octopus vulgaris*. *Front. Physiol* 8, 453.

Garrido, D., Navarro, J. C., Perales-Raya, C., Nande, M., Martín, M. V., Iglesias, J., Bartolomé, A., Roura, A., Varó, I., Otero, J. J., González, A. F., Rodríguez, C., and Almansa, E. (2016). Fatty acid composition and age estimation of wild *Octopus vulgaris* paralarvae. *Aquaculture* 464, 564−569.

Lourenço, S., Roura, A., Fernández-Reiriz, M. J., Narciso, L., and González, A. F. (2017). Feeding relationship between *Octopus vulgaris* (Cuvier, 1797) early life-cycle stages and their prey in the Western Iberian upwelling system: correlation of reciprocal lipid and fatty acid contents. *Front. Physiol*. 8, 467.

Navarro, J. C., and Villanueva, R. (2000). Lipid and fatty acid composition of early stages of cephalopods: an approach to their lipid requirements. *Aquaculture* 183, 161−177.

Navarro, J. C., and Villanueva, R. (2003). The fatty acid composition of *Octopus vulgaris* paralarvae reared with live and inert food: deviation from their natural fatty acid profile. *Aquaculture* 219, 613−631.

Supplementary Table 2. Summary of samples taken from each tissue to analyse the eight biochemical compounds. Shown are the number of records (N) and mean (SD) value for each tissue and compound combination, expressed as g/100 g dry weight (% DW) with the exception of water content. Note that triglycerides were not measured in the arm and mantle.

**Compound**	**Arm**		**Mantle**		**Ovary**		**Digestive Gland**	
	N	Mean (SD)	N	Mean (SD)	N	Mean (SD)	N	Mean (SD)
Water	246	76.8 (3.6)	246	79.7 (4.0)	129	65.5 (5.9)	246	64.9 (5.4)
Lipids	246	1.5 (0.3)	246	1.9 (0.4)	114	7.8 (2.6)	246	19.8 (7.8)
Free Fatty Acids	246	0.02 (0.01)	246	0.04 (0.03)	114	0.1 (0.08)	246	0.9 (0.5)
Triglycerides					114	1.3 (1.0)	241	9.6 (6.5)
Phospholipids	246	1.1 (0.3)	246	1.4 (0.4)	114	4.0 (1.1)	245	2.5 (1.2)
Sterols	246	0.2 (0.06)	246	0.2 (0.07)	114	0.7 (0.2)	245	1.1 (0.5)
Proteins	244	83.7 (3.9)	246	79.9 (5.9)	115	68.9 (5.3)	237	51.2 (9.4)
Glycogen	55	1.6 (1.3)	55	2.2 (2.1)	28	5.4 (1.9)	43	2.6 (0.9)

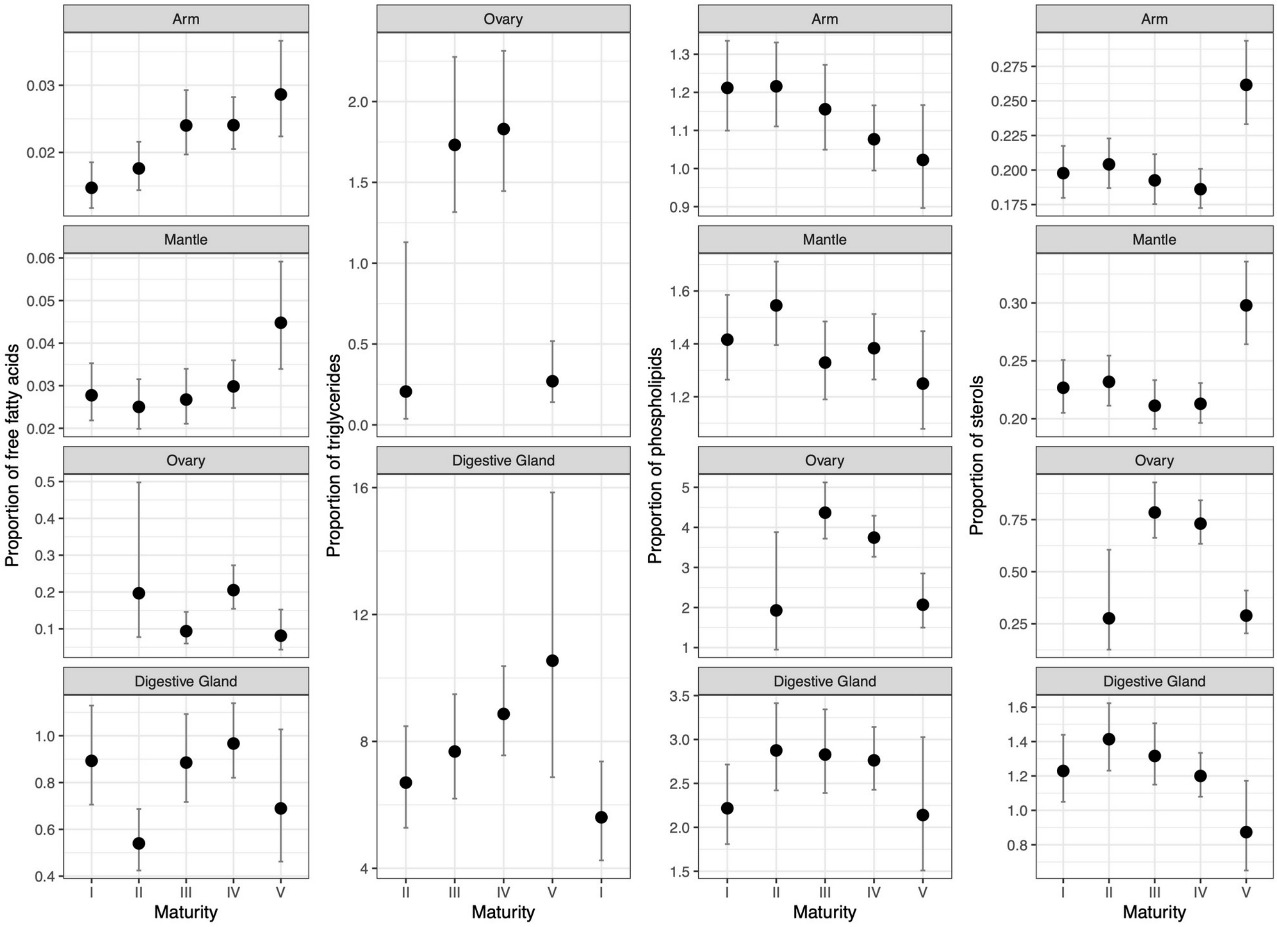

Supplementary Figure 2. Differences in the proportion of free fatty acids, triglycerides, phospholipids and sterols (across columns) per tissue (along rows) across maturity stages once accounting for seasonality (Supplementary Figure 4) and body (for arm and mantle tissues), ovary or digestive gland log-transformed weight (Supplementary Figure 6) as obtained from fitting beta regression models. Predicted values were obtained for an individual of 1.5 kg in spring. Note that triglycerides were not measured in the arm or the mantle.

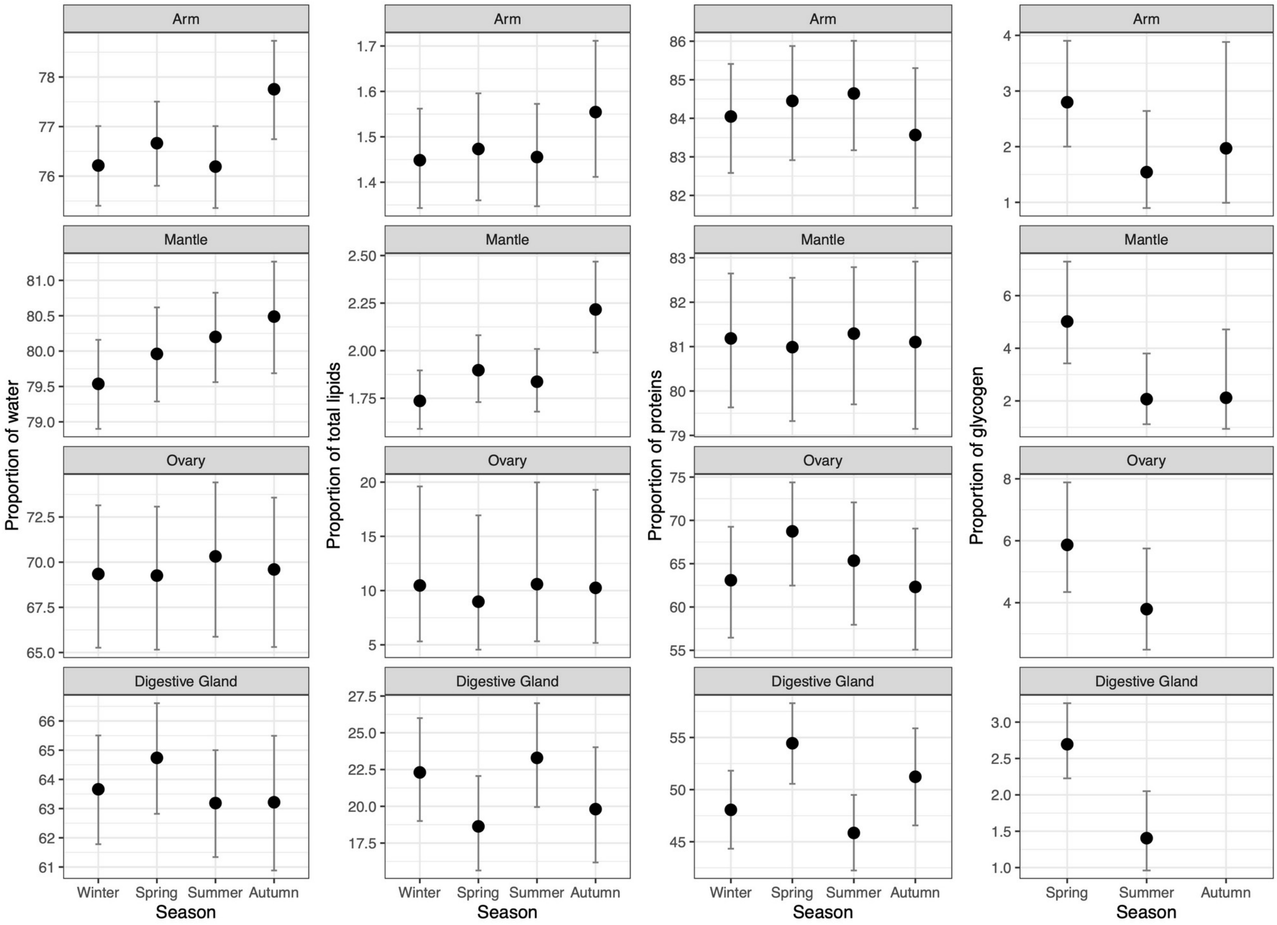

Supplementary Figure 3. Seasonal differences in the proportion of each of the four main biochemical constituents (across columns) per tissue (along rows) once accounting for maturity stage (Figure 3 in the main text) and body (for arm and mantle tissues), ovary or digestive gland log-transformed weight (Supplementary Figure 5) as obtained from fitting beta regression models. Predicted values were obtained for an individual in macrostage IV and 1.5 kg.

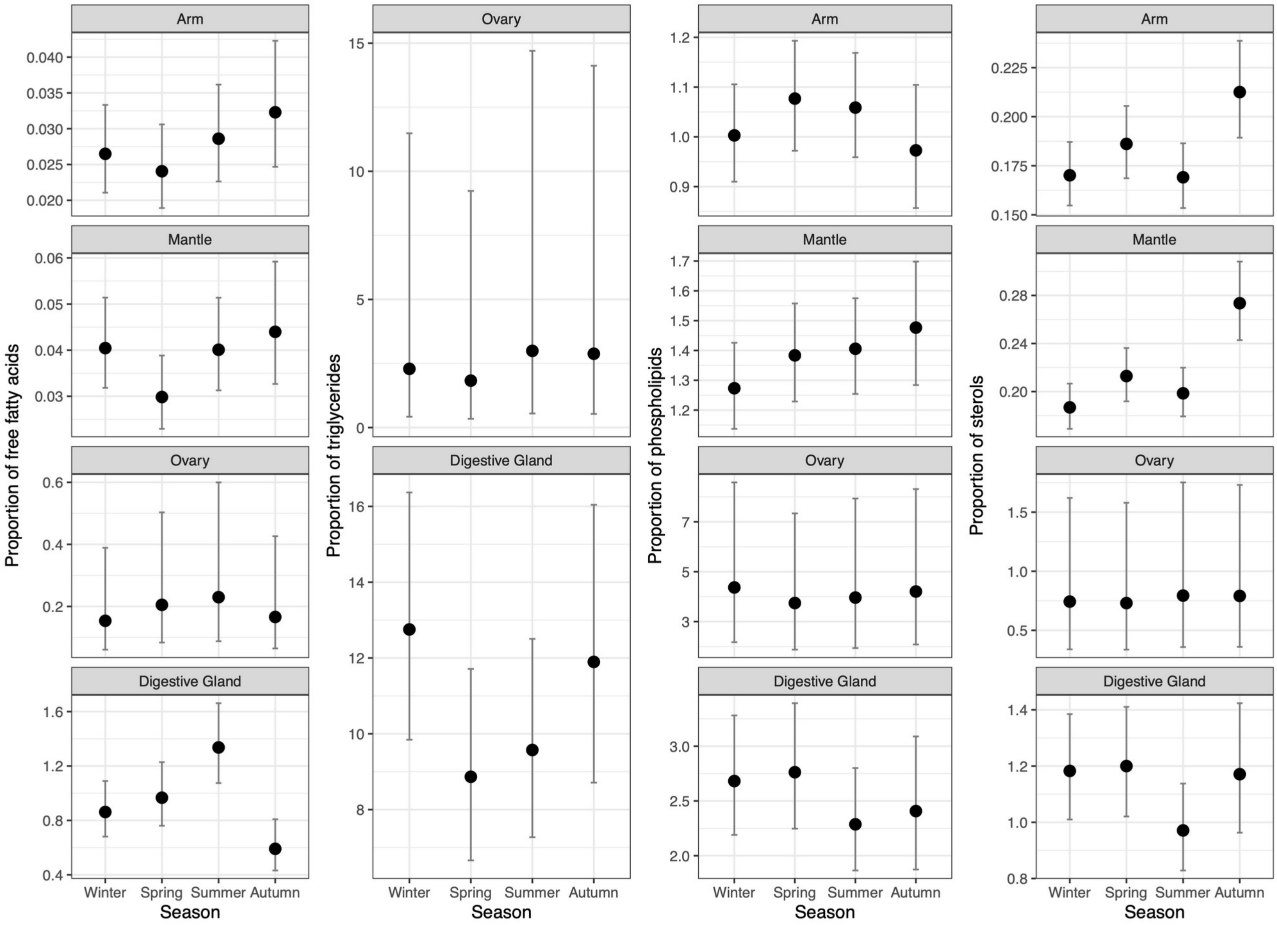

Supplementary Figure 4. Seasonal differences in the proportion of free fatty acids, triglycerides, phospholipids and sterols (across columns) per tissue (along rows) once accounting for maturity stage (Supplementary Figure 2) and body (for arm and mantle tissues), ovary or digestive gland log-transformed weight (Supplementary Figure 6) as obtained from fitting beta regression models. Predicted values were obtained for an individual in macrostage IV and 1.5 kg. Note that triglycerides were not measured in the arm or the mantle.

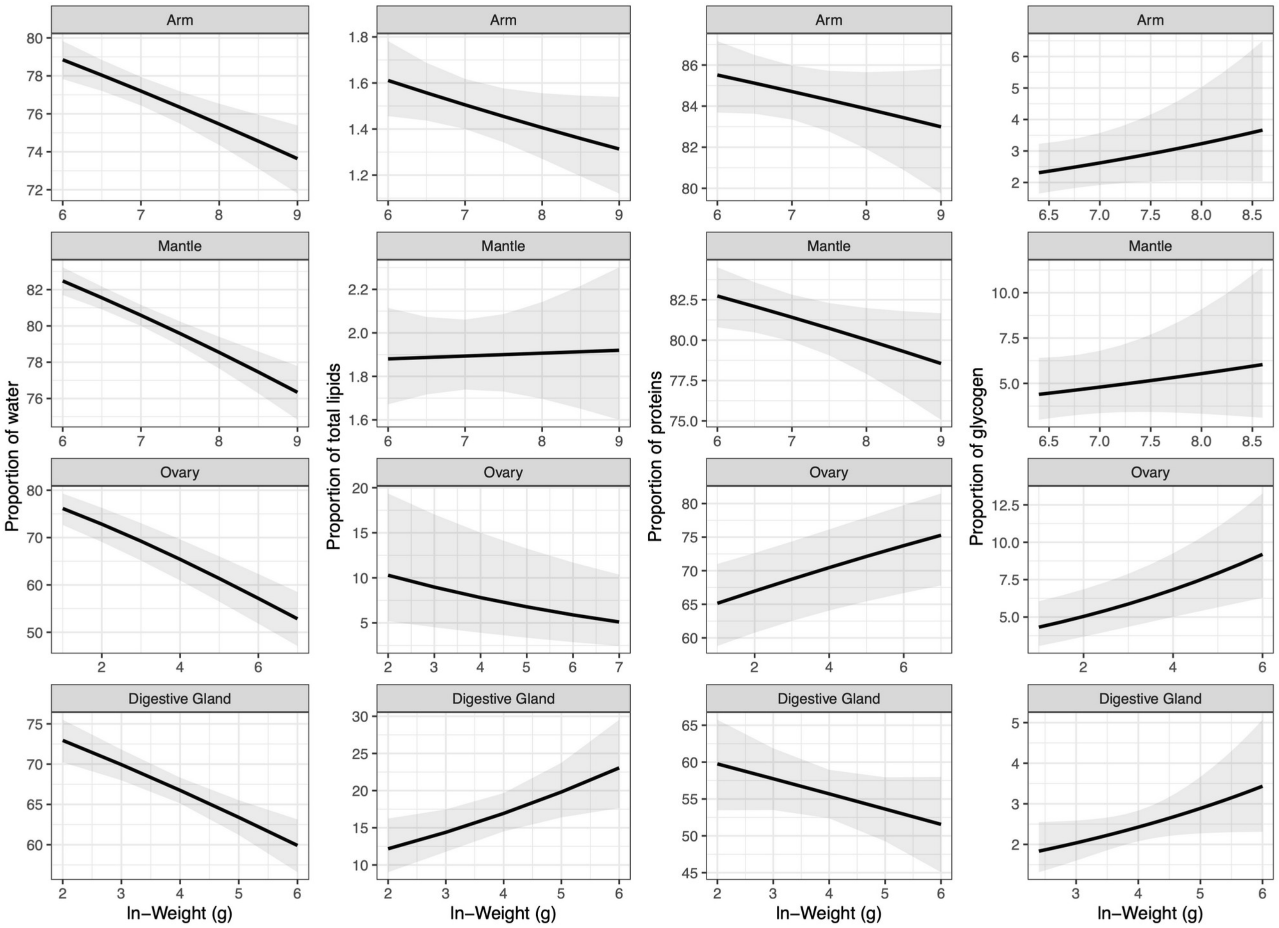

Supplementary Figure 5. Relationship between the proportion of each of the four main biochemical constituents (across columns) per tissue (along rows) with natural log-transformed body (for arm and mantle tissues), ovary or digestive gland weight once accounting for maturity stage (Figure 3 in the main text) and seasonality (Supplementary Figure 3) as obtained from fitting beta regression models. Predicted values were obtained for a macrostage IV individual in spring.

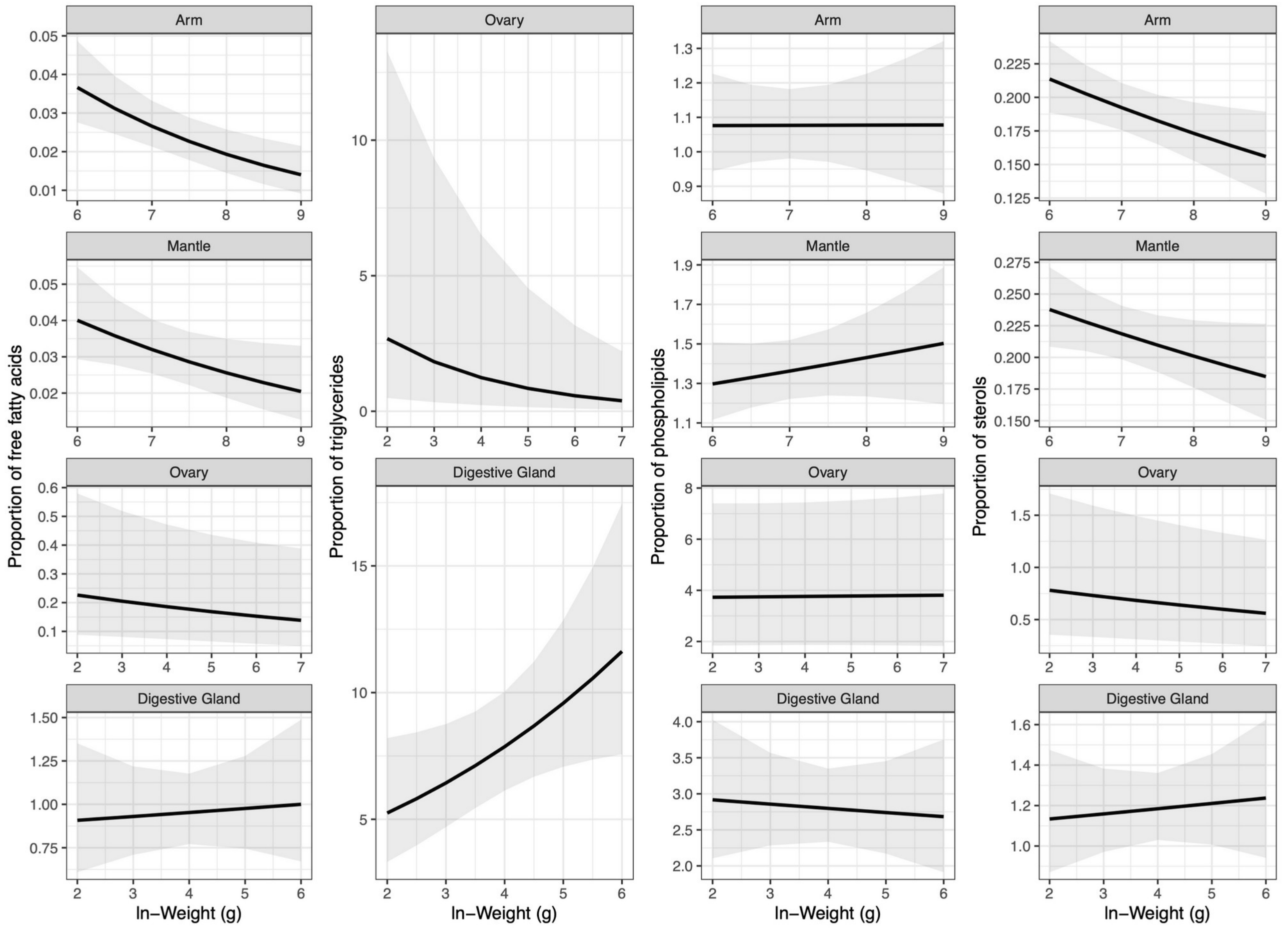

Supplementary Figure 6. Relationship between the proportion of free fatty acids, triglycerides, phospholipids and sterols (across columns) per tissue (along rows) with natural log-transformed body (for arm and mantle tissues), ovary or digestive gland weight once accounting for maturity stage (Supplementary Figure 2) and seasonality (Supplementary Figure 4) as obtained from fitting beta regression models. Predicted values were obtained for a macrostage IV individual in spring. Note that triglycerides were not measured in the arm or the mantle.

Supplementary Table 3. Numerical results showing various models' equations for different bivariate relationships shown in the main text. Note that, for simplicity, only the intercept and slope are provided. For GAMs, that do not have a simple equation, the estimated degrees of freedom (Edf) are provided. Finally, for quantile regressions, just equations for 0.1, 0.5 and 0.9 quantiles are provided.

Modelling purpose	Model type	Equation	Figure
Compounds in Digestive Gland: Maturity I	Beta regression	y = 1.09 − 2.29 x	Fig. 4A
Compounds in Digestive Gland: Maturity II	Beta regression	y = 0.94 − 1.62 x	Fig. 4A
Compounds in Digestive Gland: Maturity III	Beta regression	y = 0.90 − 1.57 x	Fig. 4A
Compounds in Digestive Gland: Maturity IV	Beta regression	y = 0.64 − 0.62 x	Fig. 4A
Compounds in Digestive Gland: Maturity V	Beta regression	y = 1.38 − 2.44 x	Fig. 4A
Compounds in Digestive Gland: Maturity I	Beta regression	y = −0.10 + 1.56 x	Fig. 4B
Compounds in Digestive Gland: Maturity II	Beta regression	y = 0.09 + 1.05 x	Fig. 4B
Compounds in Digestive Gland: Maturity III	Beta regression	y = 0.19 + 0.72 x	Fig. 4B
Compounds in Digestive Gland: Maturity IV	Beta regression	y = 0.38 + 0.25 x	Fig. 4B
Compounds in Digestive Gland: Maturity V	Beta regression	y = −0.43 + 2.79 x	Fig. 4B
Compounds in Digestive Gland: Maturity I	Beta regression	y = 0.72 − 3.60 x	Fig. 4C
Compounds in Digestive Gland: Maturity II	Beta regression	y = 1.06 − 5.11 x	Fig. 4C
Compounds in Digestive Gland: Maturity III	Beta regression	y = 0.87 − 3.88 x	Fig. 4C
Compounds in Digestive Gland: Maturity IV	Beta regression	y = 1.18 − 5.38 x	Fig. 4C
Compounds in Digestive Gland: Maturity V	Beta regression	y = 0.64 − 3.75 x	Fig. 4C
Compounds in Ovary: Maturity IV	Beta regression	y = 0.09 + 5.62 x	Fig. 4D
Compounds in Ovary: Maturity IV	Beta regression	y = 2.16 − 2.35 x	Fig. 4E
Compounds in Ovary: Maturity IV	Beta regression	y = 1.05 − 3.59 x	Fig. 4F
Compounds in Arm: Maturity V	Beta regression	y = 6.10 − 5.38 x	Fig. 4G
Compounds in Mantle: Maturity V	Beta regression	y = 3.48 − 1.90 x	Fig. 4H
Compounds in Ovary: Maturity V	GAM	Edf = 1	Fig. 4I (inset)
Compounds in Ovary: Maturity V	GAM	Edf = 2.26	Fig. 4I
Lipids and energy density in arm	Beta regression	y = −3.97 + 0.03 x	Fig. 9A
Proteins and energy density in arm	Beta regression	y = 3.61 − 0.03 x	Fig. 9B
Glycogen and energy density in arm	Beta regression	y = −4.57 + 0.01 x	Fig. 9C
Lipids and energy density in mantle	Beta regression	y = −2.76 − 0.02 x	Fig. 9D
Proteins and energy density in mantle	Beta regression	y = 2.88 − 0.01 x	Fig. 9E
Glycogen and energy density in mantle	Beta regression	y = −4.82 + 0.05 x	Fig. 9F

Lipids and energy density in ovary	Beta regression	y = −6.65 + 0.23 x	Fig. 9G
Proteins and energy density in ovary	Beta regression	y = 4.40 − 0.14 x	Fig. 9H
Glycogen and energy density in ovary	Beta regression	y = −1.57 − 0.07 x	Fig. 9I
Lipids and energy density in digestive gland	Beta regression	y = −5.05 + 0.22 x	Fig. 9J
Proteins and energy density in digestive gland	Beta regression	y = 4.71 − 0.21 x	Fig. 9K
Glycogen and energy density in digestive gland	Beta regression	y = −2.86 − 0.05 x	Fig. 9L
Ovary energy versus digestive gland energy	Quantile regression (τ = 0.1, 0.5, 0.9)	y = 69.78 + 0.08 x<br>y = 290.85 + 0.33 x<br>y = 856.65 + 1.02 x	Fig. 10D
Ovary energy versus muscle energy	Quantile regression (τ = 0.1, 0.5, 0.9)	y = 205.76 − 0.01 x<br>y = 308.89 + 0.03 x<br>y = 1355.31 + 0.09 x	Fig. 10E
Digestive gland energy versus muscle energy	Quantile regression (τ = 0.1, 0.5, 0.9)	y = −75.85 + 0.06 x<br>y = −128.54 + 0.10 x<br>y = 163.33 + 0.13 x	Fig. 10F

Supplementary Table 4. Fatty acid composition (% of total fatty acids), total FAME (% DW), and total lipids (% DW) in each tissue of female *O. vulgaris* expressed as mean ± SD. Only those fatty acids with concentrations ≥1% are shown.

**Compound**	**Arm**	**Mantle**	**Ovary**	**Digestive Gland**
14:0	1.08±0.70	0.92±0.67	2.30±1.61	2.29±1.53
16:0	12.90±7.45	12.76±7.37	13.85±9.03	10.38±5.38
16:1n7	0.57±0.41	0.59±0.40	0.58±0.65	4.31±2.66
17:0	1.08±0.69	1.20±0.72	0.79±0.60	1.14±0.66
17:1	2.20±1.49	2.04±1.32	1.40±1.13	0.97±0.86
18:0	5.86±3.64	6.63±4.23	3.63±2.69	5.45±3.24
18:1n9c	1.79±1.23	1.86±1.22	2.03±1.56	5.50±3.39
18:1n7	1.21±0.74	1.22±0.73	1.02±0.71	3.26±1.89
20:1n9	3.82±1.21	3.65±1.34	5.67±1.66	3.04±1.65
20:2	0.50±0.73	0.51±0.34	0.40±0.33	1.53±1.35
20:4n6	6.05±2.68	6.53±2.80	10.05±4.50	7.84±4.03
20:5n3	21.25±4.88	21.10±5.02	17.24±4.35	19.51±7.42
22:1n9	1.29±0.77	1.11±0.72	0.53±0.42	1.53±1.64
22:5n3	2.16±1.74	1.67±0.97	1.64±1.08	2.60±1.58
24:0	1.09±1.55	1.14±2.38	1.45±2.57	1.74±3.67
22:6n3	36.34±8.84	36.40±9.20	36.08±10.26	26.44±10.93
ΣSAFA	22.33±11.96	22.91±12.04	22.40±13.47	21.96±10.00
ΣMUFA	11.20±2.64	10.68±2.62	12.00±2.99	19.16±6.21
ΣPUFA	66.47±14.06	66.41±14.13	65.60±15.86	58.88±15.01
ΣHUFA	65.81±14.09	65.71±14.24	65.09±15.92	56.65±14.89
Σn-3	59.76±13.16	59.18±13.54	55.04±13.34	48.81±14.28
Σn-6	6.21±2.67	6.72±2.78	10.16±4.46	8.53±3.93
Σn-3/Σn-6	11.01±4.30	10.13±4.22	6.31±2.69	7.03±3.76
DHA/EPA	1.73±0.28	1.74±0.28	2.13±0.54	1.52±0.81
DHA/ARA	6.88±2.72	6.42±2.64	4.11±1.64	4.56±3.49
EPA/ARA	4.09±1.75	3.81±1.81	2.09±1.04	3.12±1.75
ΣFAME	0.79±0.28	1.02±0.70	4.74±2.21	11.80±7.03
Total lipids	1.52±0.31	1.95±0.44	7.85±2.57	19.79±7.79

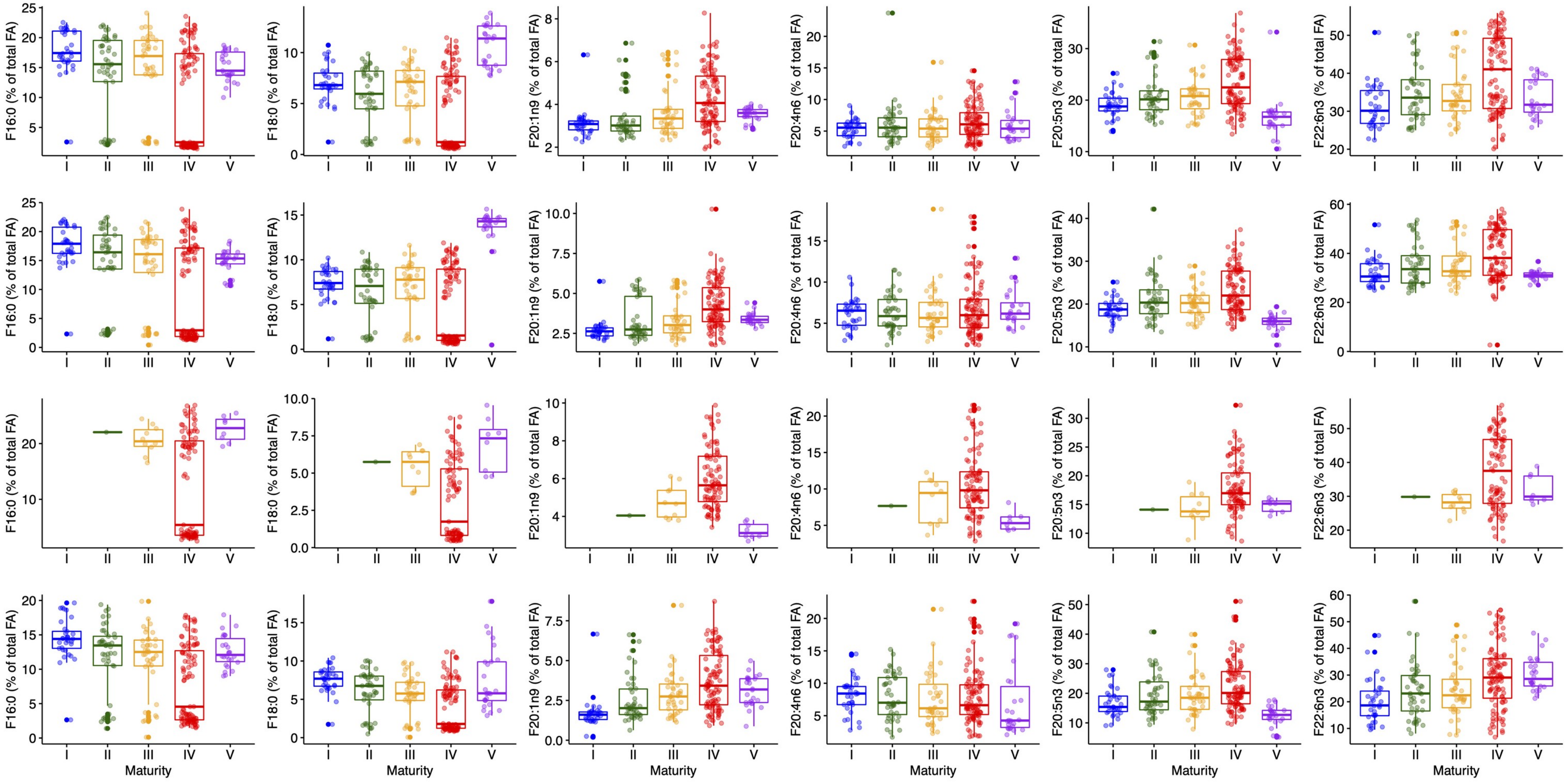

Supplementary Figure 7. Box and whisker plots showing the distribution of data for specific fatty acids among maturity macrostages in the arm (first row), mantle (second row), ovary (third row) and digestive gland (fourth row).

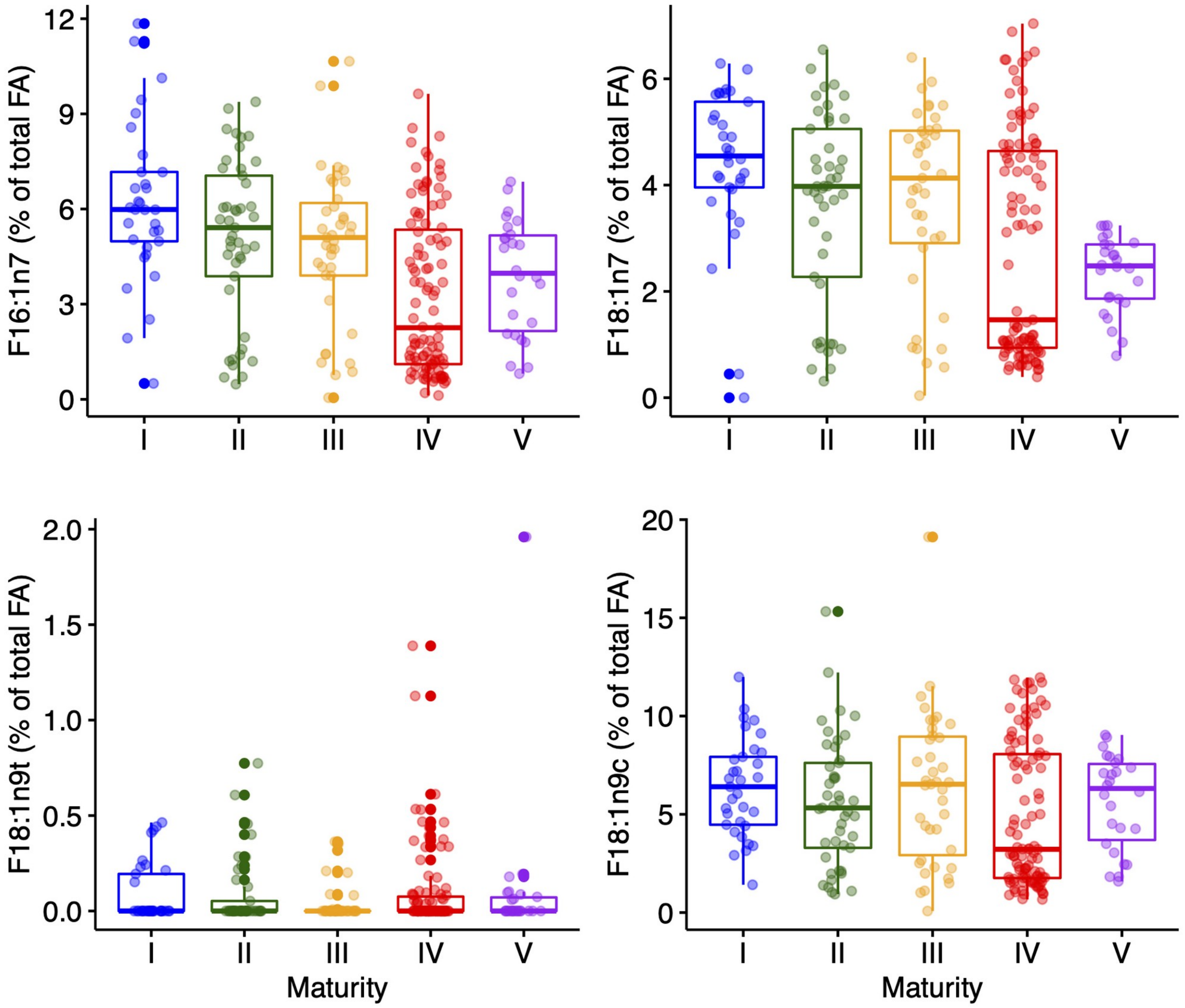

Supplementary Figure 8. Box and whisker plots showing the distribution of data for specific fatty acids among maturity stages in the digestive gland not shown in the previous figure.

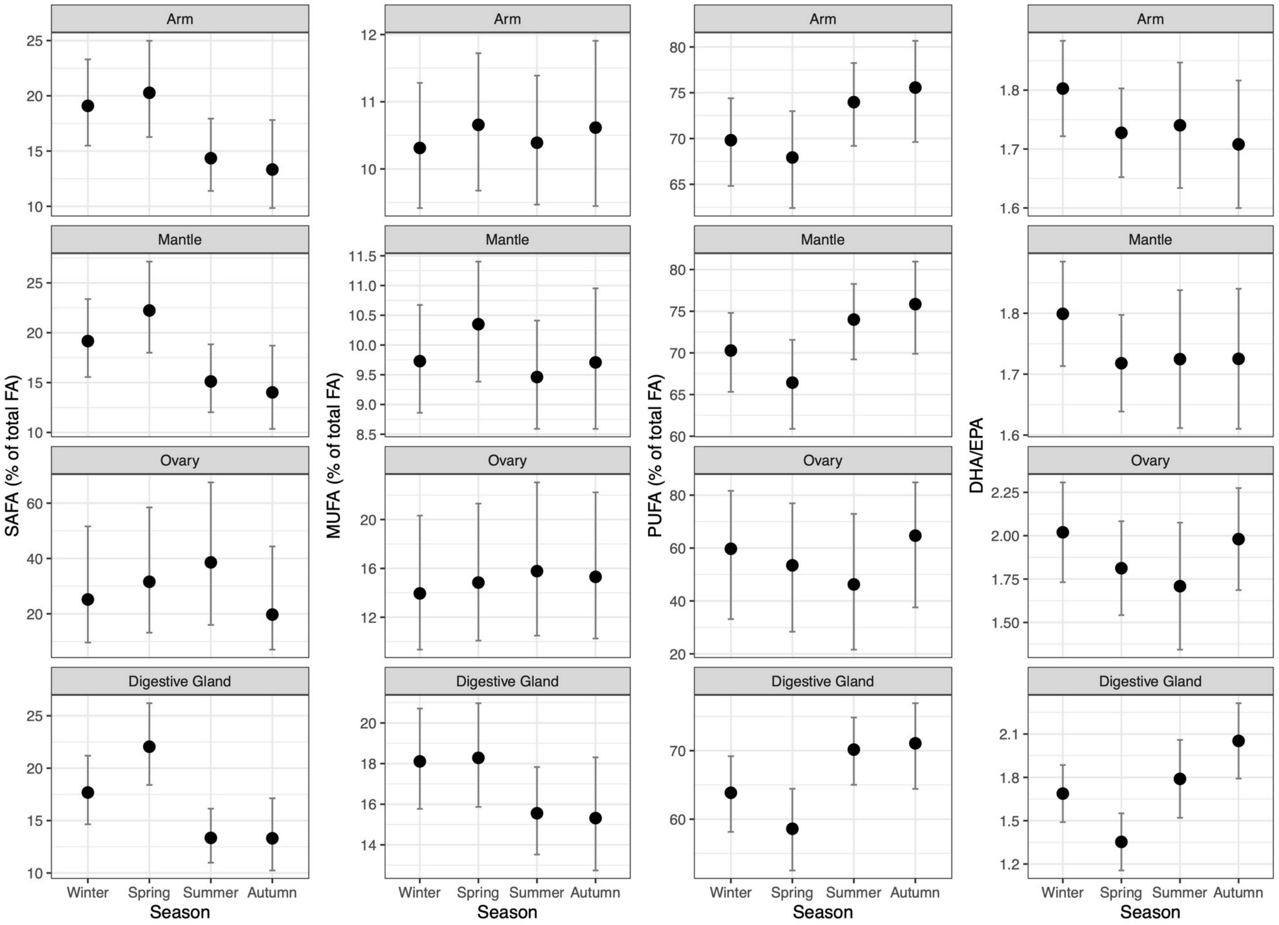

Supplementary Figure 9. Seasonal differences in the proportion of fatty acid groups and DHA/EPA ratio (across columns) per tissue (along rows) once accounting for maturity stage (Figure 7 in the main text) and body (for arm and mantle tissues), ovary or digestive gland log-transformed weight (Supplementary Figure 10) as obtained from fitting beta regression models. Predicted values were obtained for an individual in macrostage IV and 1.5 kg.

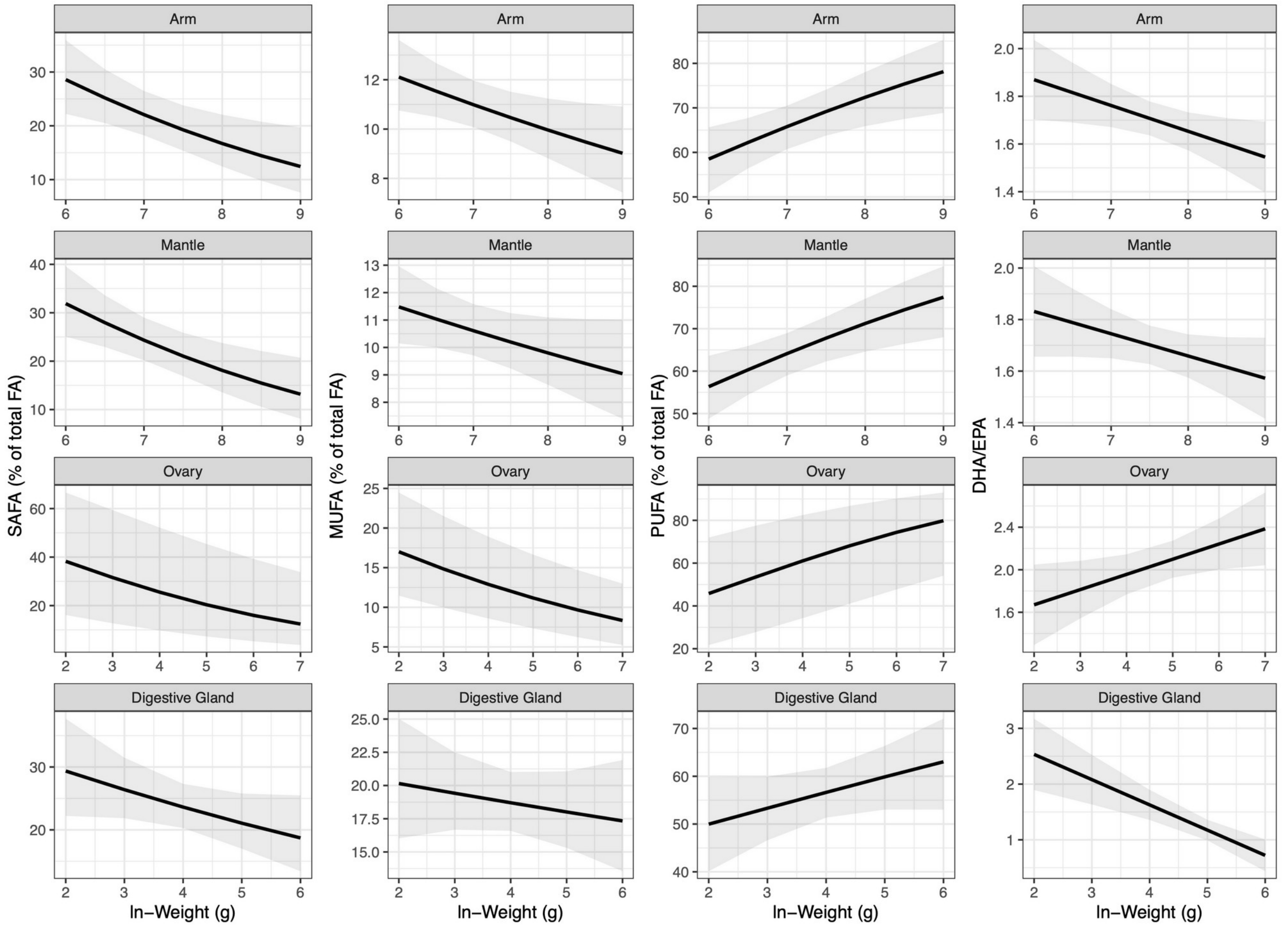

Supplementary Figure 10. Relationship between the proportion of fatty acid groups and DHA/EPA ratio (across columns) per tissue (along rows) with natural log-transformed body (for arm and mantle tissues), ovary or digestive gland weight once accounting for maturity stage (Figure 7 in the main text) and seasonality (Supplementary Figure 9) as obtained from fitting beta regression models. Predicted values were obtained for an individual in macrostage IV in spring.

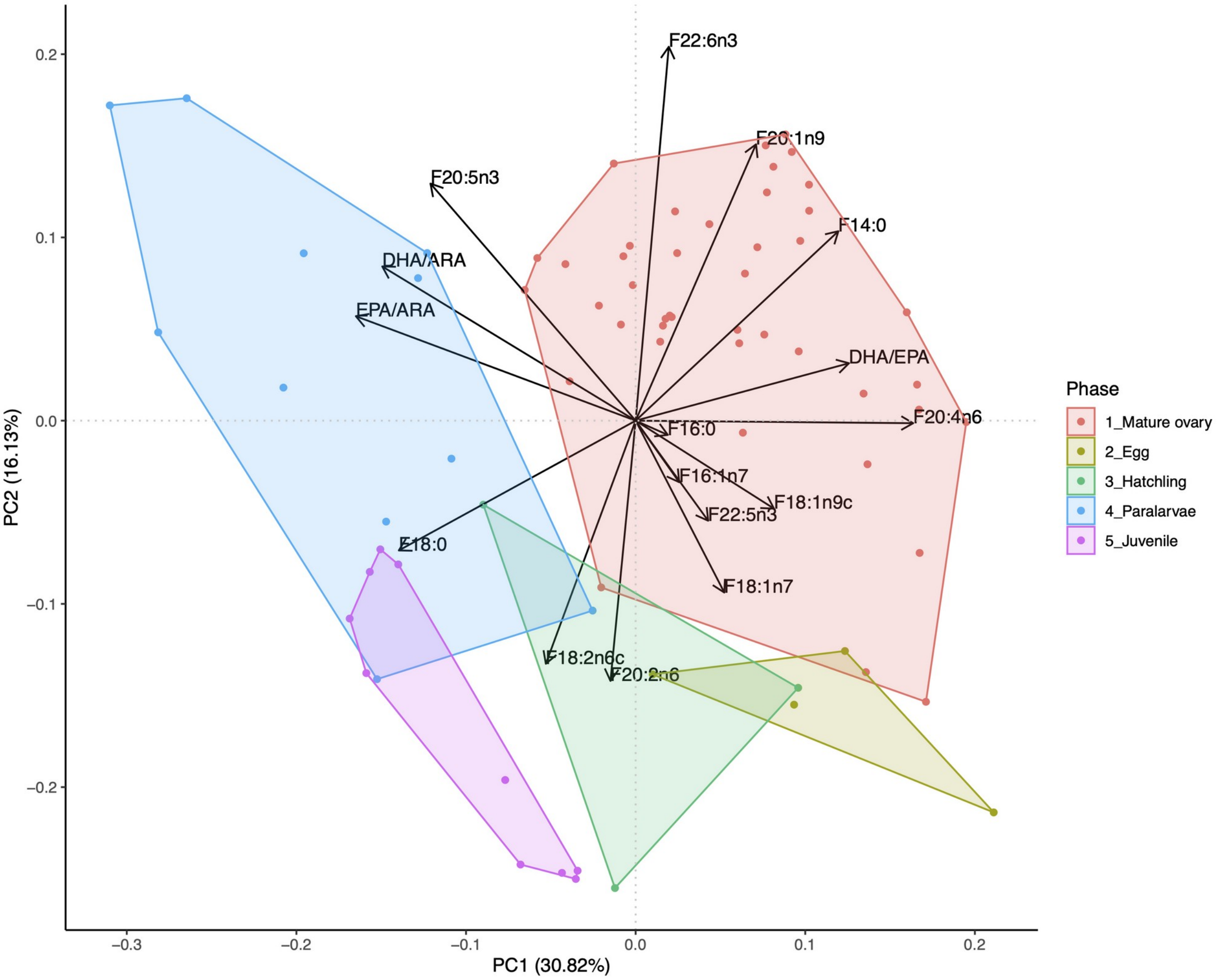

Supplementary Figure 11. Principal component analysis showing the contribution of each analysed fatty acid from mature ovaries studied in this work as compared to data obtained from the literature (Supplementary Table 1) for different wild life stages. Note that, for the case of our mature ovaries, this PCA excludes year 2005 (see Figure 8 in the main text for a PCA including 2005).

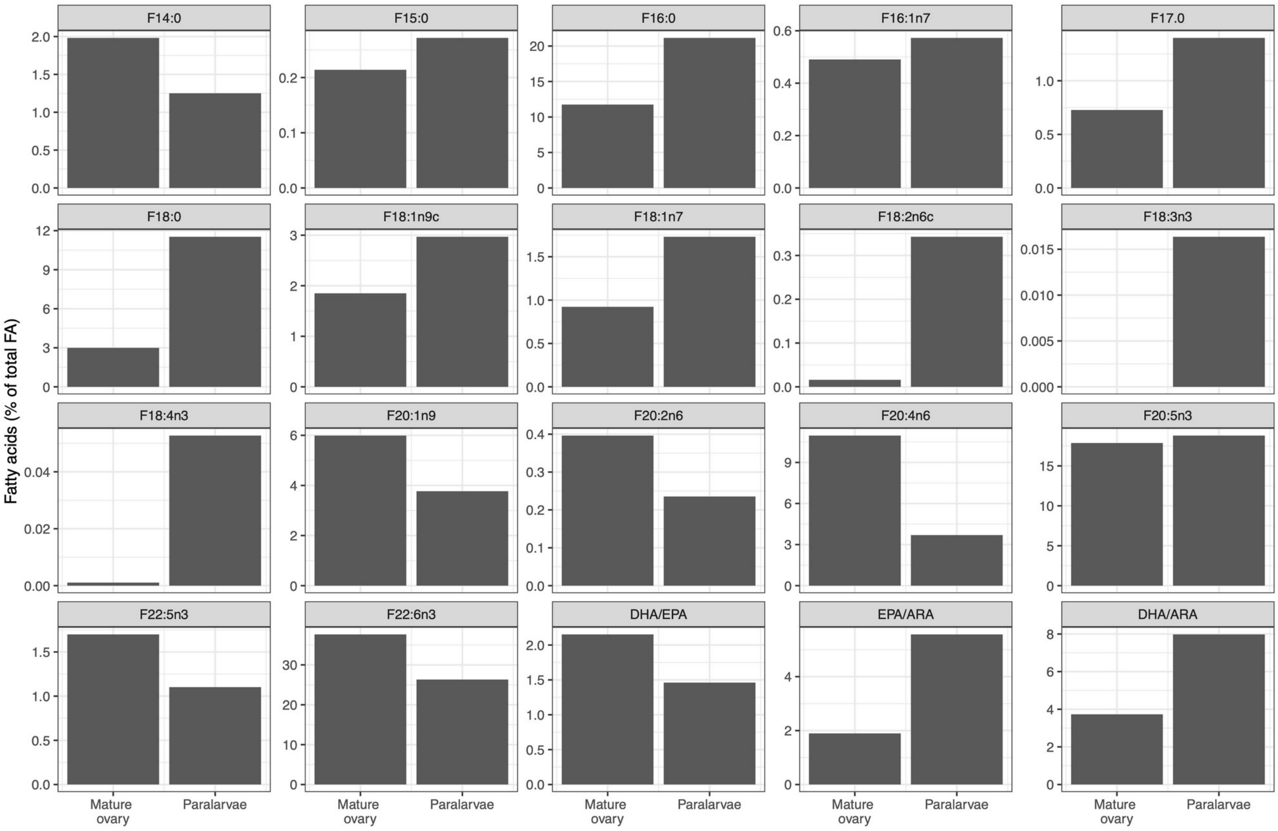

Supplementary Figure 12. Average percentage of specific fatty acids in the mature ovaries studied here and in wild paralarvae sourced from the literature.

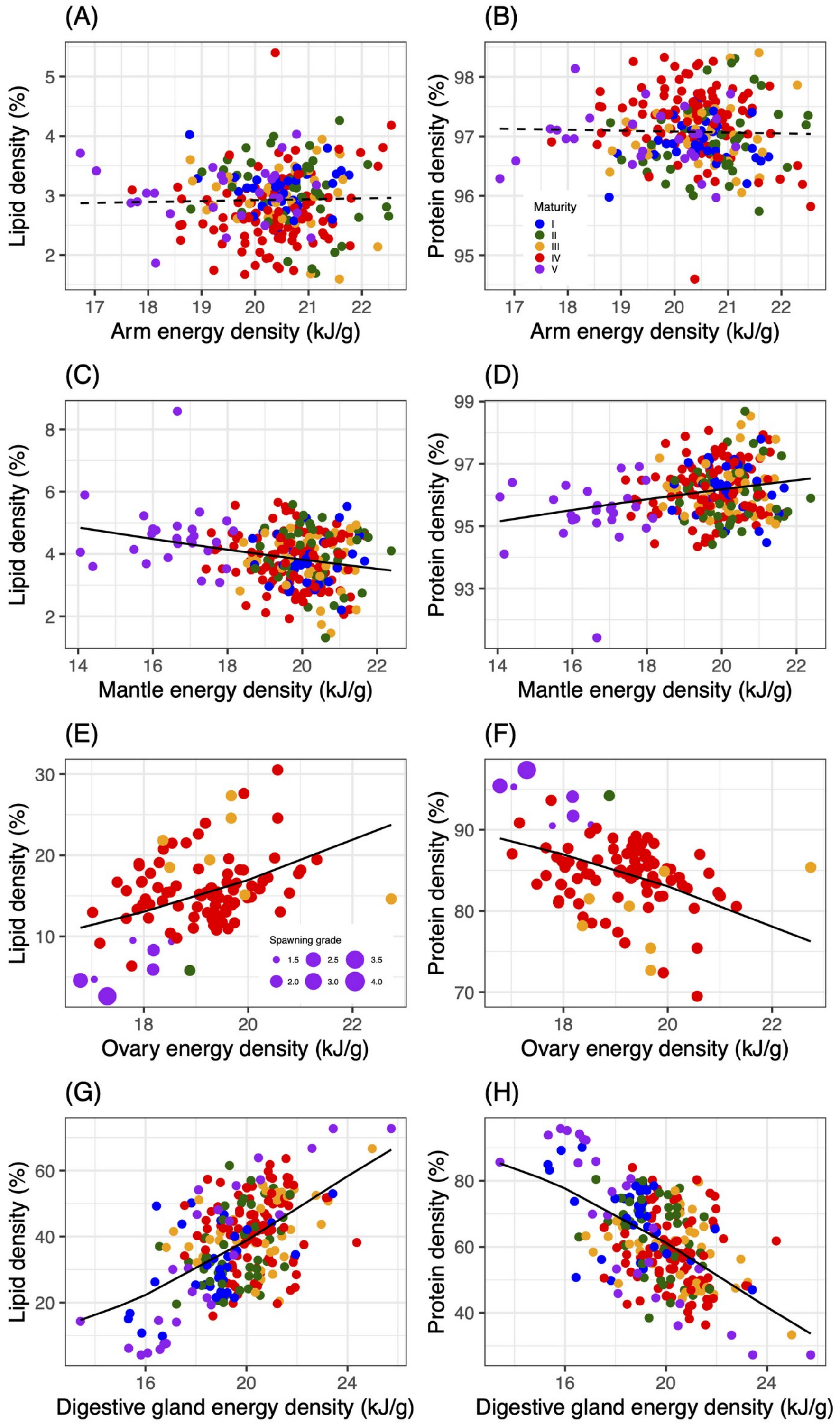

Supplementary Figure 13. Same as Figure 9 in the main text but excluding glycogen from the calculations of density content. Lines show beta regression fits with solid lines indicating that the slope is statistically significant ($p < 0.05$).

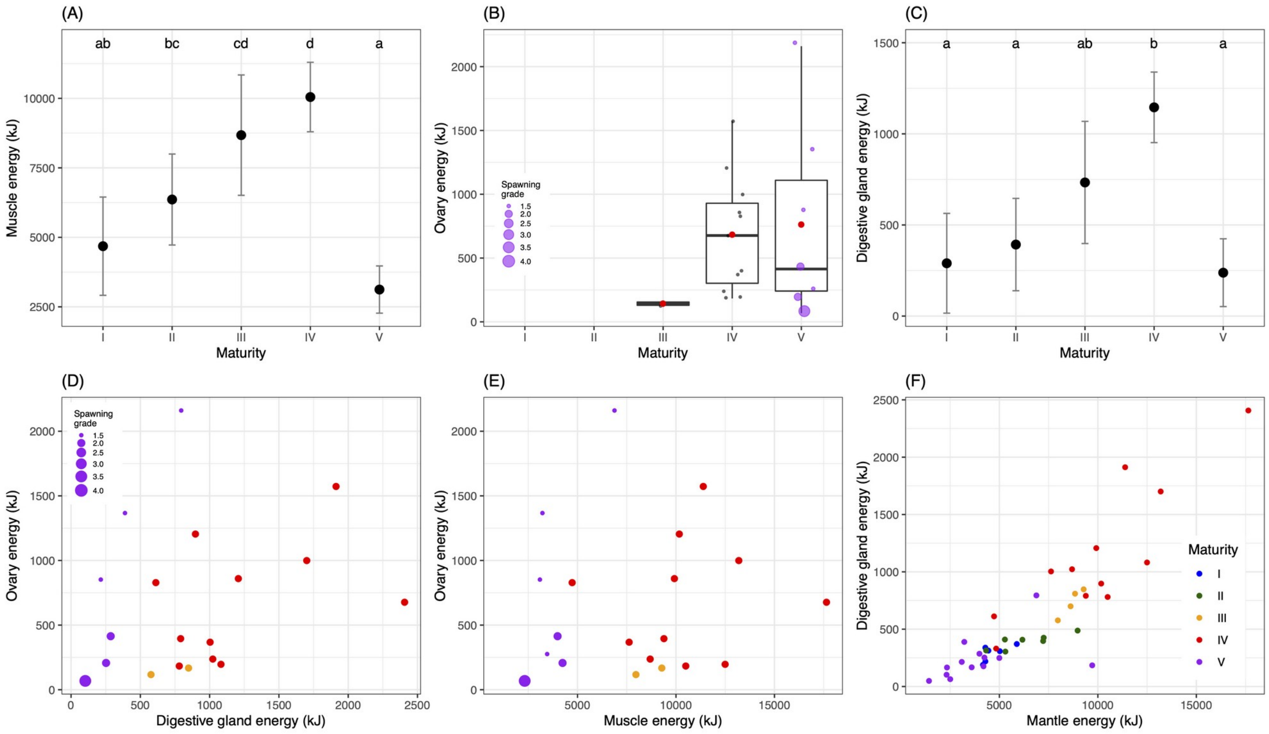

Supplementary Figure 14. Same as Figure 10 in the main text but including glycogen in the calculations of energy content.
